# Supplementary material for: Model-based prediction of spatial gene expression via generative linear mapping
Source: Nat Commun. 2021 Jun 17;12:3731. doi: 10.1038/s41467-021-24014-x (PMC8211835; doi:10.1038/s41467-021-24014-x)
Supplement: Supplementary file 1 — Supplementary Information [file 41467_2021_24014_MOESM1_ESM.pdf]

Supplementary Information

Model-based Prediction of Spatial Gene Expression via Generative Linear Mapping

Yasushi Okochi[a,b]†, Shunta Sakaguchi[c]†, Ken Nakae[d], Takefumi Kondo[c,e], Honda Naoki[a,f, g]*

[a] Laboratory for Theoretical Biology, Graduate School of Biostudies, Kyoto University, Sakyo, Kyoto, Kyoto, Japan

[b] Faculty of Medicine, Kyoto University, Sakyo, Kyoto, Kyoto, Japan

[c] Laboratory for Cell Recognition and Pattern Formation, Graduate School of Biostudies, Kyoto University, Sakyo, Kyoto, Kyoto, Japan

[d] Graduate School of Informatics, Kyoto University, Sakyo, Kyoto, Kyoto, Japan

[e] The Keihanshin Consortium for Fostering the Next Generation of Global Leaders in Research (K-CONNEX), Sakyo, Kyoto, Kyoto, Japan

[f] Laboratory for Data-driven Biology, Graduate School of Integrated Sciences for Life, Hiroshima University, Higashihiroshima, Hiroshima, Japan

[g] Theoretical Biology Research Group, Exploratory Research Center on Life and Living Systems (ExCELLS), National Institutes of Natural Sciences, Okazaki, Aichi, Japan.

† These authors contributed equally to this manuscript.

*** Corresponding author**.

Honda Naoki

Graduate School of Integrated Sciences for Life, Hiroshima University, Kagagamiya 1-3-1, Higashihiroshima, Hiroshima, 739-8526, Japan

Tel.: +81-82-424-7336

E-mail: nhonda@hiroshima-u.ac.jp

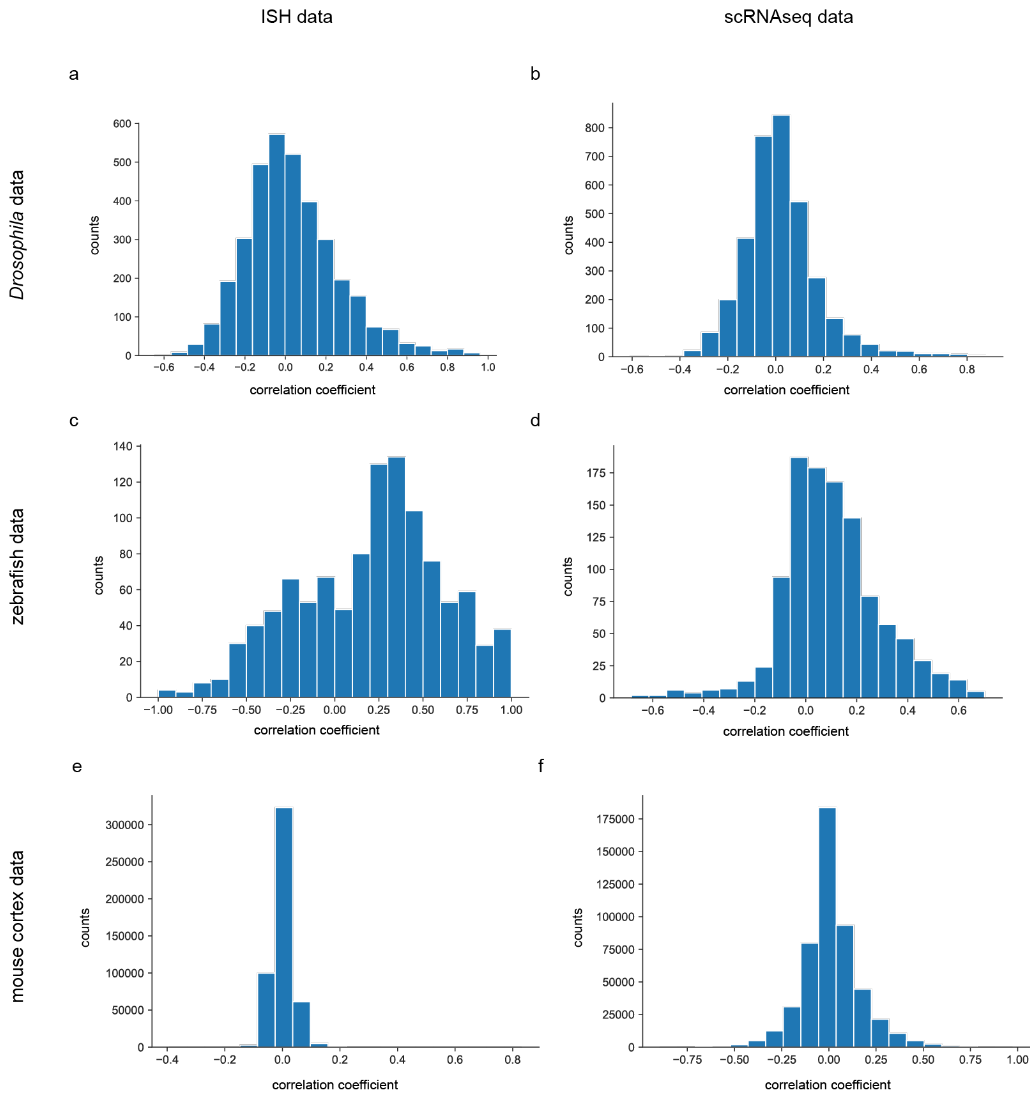

Supplementary Figure 1: Gene correlation in the ISH and the scRNA-seq data

Pearson's correlation coefficients between all pairs of landmark genes for the ISH data (a, c, e) and scRNA-seq data (b, d, f) of the Drosophila dataset (a, b), zebrafish dataset (c, d) and mouse cortex dataset (e, f).

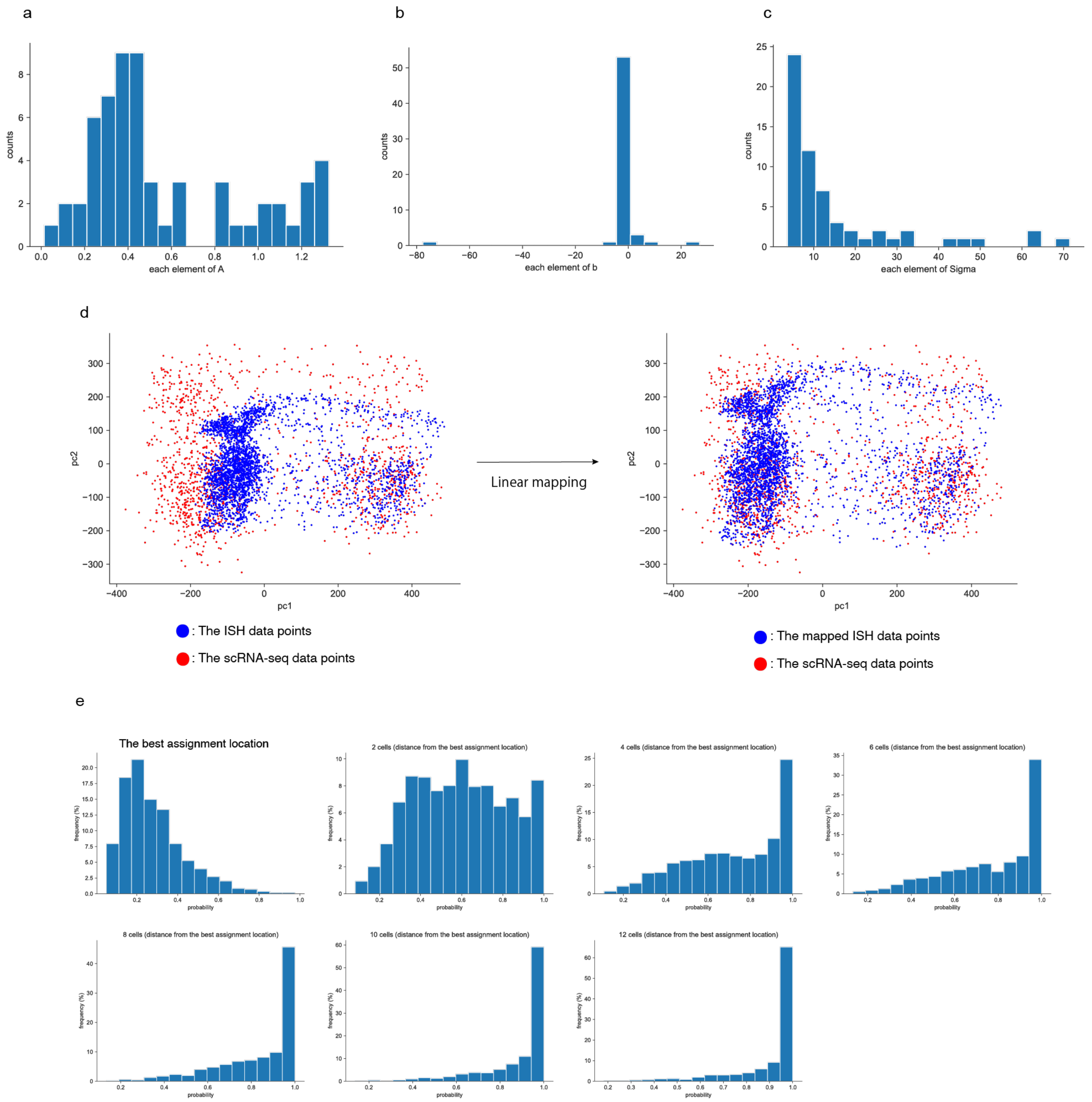

Supplementary Figure 2: Linear mapping property of Perler

(a–c) Histograms of the distributions of the estimated parameters of generative linear mapping: A (left), b (middle), and Σ (right) (see Methods). Note that because A and Σ are diagonal matrices, only the diagonal elements of A and Σ are shown in the middle and right panels. (d) Scatter plot of scRNA-seq and ISH data points before (left) and after (right) mapping and corresponding to Fig. 1b and Fig. 2a. Principal component analysis[14] was used to visualize high-dimensional gene-expression data into two dimensions. (e) Histograms of the assigned confidence corresponding to Fig. 2d. Each histogram shows the detailed distributions of each boxplot in Fig. 2d. Parameters of Perler are listed in Supplementary Table 7.

a

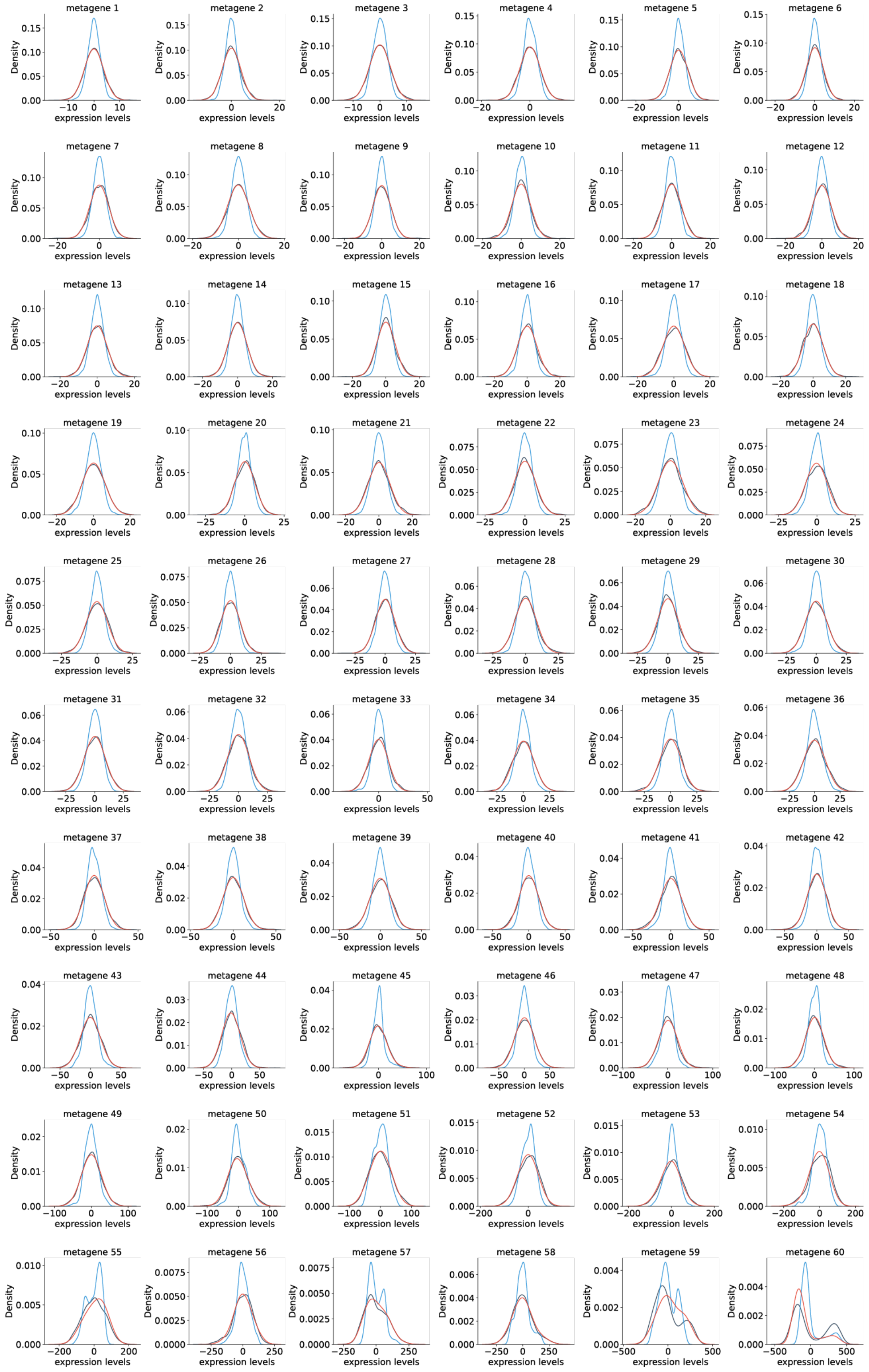
metagene 1
metagene 2
metagene 3
metagene 4
metagene 5
metagene 6
metagene 7
metagene 8
metagene 9
metagene 10
metagene 11
metagene 12
metagene 13
metagene 14
metagene 15
metagene 16
metagene 17
metagene 18
metagene 19
metagene 20
metagene 21
metagene 22
metagene 23
metagene 24
metagene 25
metagene 26
metagene 27
metagene 28
metagene 29
metagene 30
metagene 31
metagene 32
metagene 33
metagene 34
metagene 35
metagene 36
metagene 37
metagene 38
metagene 39
metagene 40
metagene 41
metagene 42
metagene 43
metagene 44
metagene 45
metagene 46
metagene 47
metagene 48
metagene 49
metagene 50
metagene 51
metagene 52
metagene 53
metagene 54
metagene 55
metagene 56
metagene 57
metagene 58
metagene 59
metagene 60
Density
expression levels

b

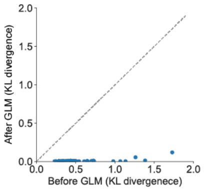
After GLM (KL divergence)
Before GLM (KL divergenece)
0.0
0.5
1.0
1.5
2.0

Supplementary Figure 3: Generative linear mapping on each metagene level for the *Drosophila* data

Comparison of distribution differences for each metagene expression level between the ISH and scRNA-seq data and those between the mapped ISH and scRNA-seq data in the Drosophila dataset. (a) Kernel density estimation of each metagene expression level in the ISH (Blue line), mapped ISH (Red line), and scRNA-seq data (Black line). For the band width parameters of the kernel density estimation in mapped ISH data, the estimated noise parameter (c_i in equation (1)) was used. (b) Scatter plot for the distribution difference. Each dot indicates the distribution difference calculated by Kullback-Leibler divergence between the ISH or the mapped ISH data and the scRNA-seq data for each metagene. Grey dashed line depicts an auxiliary line showing the same Kullback-Leibler divergence before and after the generative linear mapping. GLM, generative linear mapping. Parameters of Perler are listed in Supplementary Table 7.

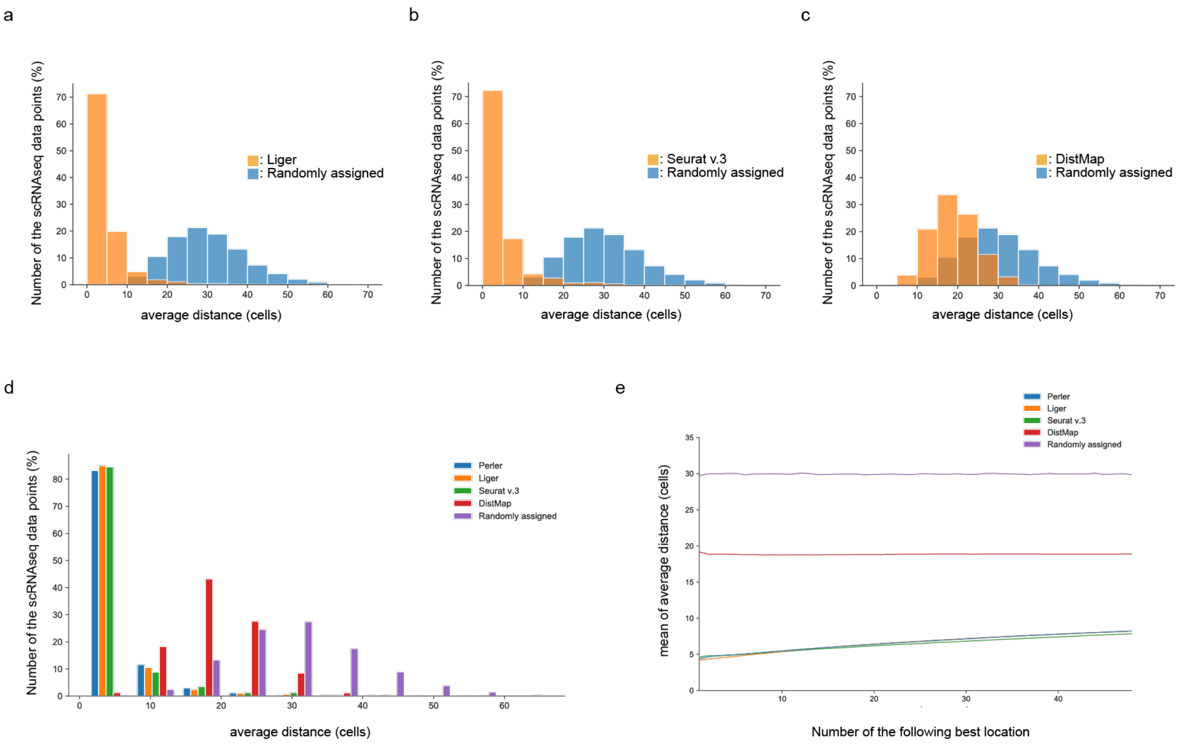

Supplementary Figure 4: Comparison of origin prediction for scRNA-seq data

(a-c) Histograms for the assigned specificity (related to Figure 2c) of other methods (Liger, Seurat v.3, and DistMap). The assigned specificity was evaluated by the distance between the best assigned location and the following best three locations. (d) Merged histogram of Figure 2c and (a-c). (e) Comparison of the assigned specificity evaluated using the different number of the following locations. Parameters of Perler are listed in Supplementary Table 7. Note, although the same analysis was performed in Karaiskos *et al.*, we generated worse results than original on our usage of DistMap.

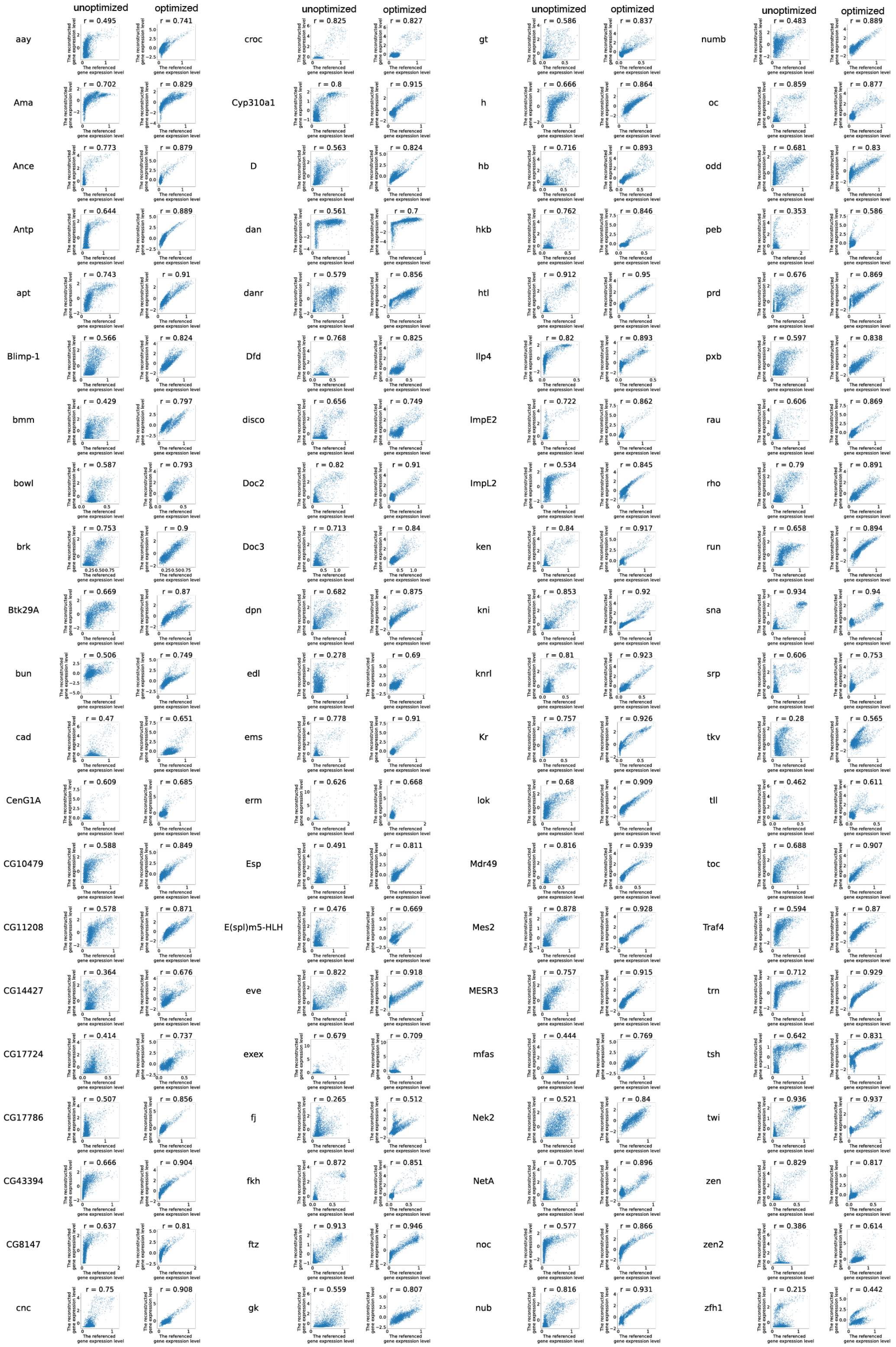

unoptimized
optimized
aay r = 0.495 r = 0.741
Ama r = 0.702 r = 0.829
Ance r = 0.773 r = 0.879
Antp r = 0.644 r = 0.889
apt r = 0.743 r = 0.91
Blimp-1 r = 0.566 r = 0.824
bmm r = 0.429 r = 0.797
bowl r = 0.587 r = 0.793
brk r = 0.753 r = 0.9
Btk29A r = 0.669 r = 0.87
bun r = 0.506 r = 0.749
cad r = 0.47 r = 0.651
CenG1A r = 0.609 r = 0.685
CG10479 r = 0.588 r = 0.849
CG11208 r = 0.578 r = 0.871
CG14427 r = 0.364 r = 0.676
CG17724 r = 0.414 r = 0.737
CG17786 r = 0.507 r = 0.856
CG43394 r = 0.666 r = 0.904
CG8147 r = 0.637 r = 0.81
cnc r = 0.75 r = 0.908
croc r = 0.825 r = 0.827
Cyp310a1 r = 0.8 r = 0.915
D r = 0.563 r = 0.824
dan r = 0.561 r = 0.7
danr r = 0.579 r = 0.856
Dfd r = 0.768 r = 0.825
disco r = 0.656 r = 0.749
Doc2 r = 0.82 r = 0.91
Doc3 r = 0.713 r = 0.84
dpn r = 0.682 r = 0.875
edl r = 0.278 r = 0.69
ems r = 0.778 r = 0.91
erm r = 0.626 r = 0.668
Esp r = 0.491 r = 0.811
E(spl)m5-HLH r = 0.476 r = 0.669
eve r = 0.822 r = 0.918
exex r = 0.679 r = 0.709
fj r = 0.265 r = 0.512
fkh r = 0.872 r = 0.851
ftz r = 0.913 r = 0.946
gk r = 0.559 r = 0.807
gt r = 0.586 r = 0.837
h r = 0.666 r = 0.864
hb r = 0.716 r = 0.893
hkb r = 0.762 r = 0.846
htl r = 0.912 r = 0.95
Ilp4 r = 0.82 r = 0.893
ImpE2 r = 0.722 r = 0.862
ImpL2 r = 0.534 r = 0.845
ken r = 0.84 r = 0.917
kni r = 0.853 r = 0.92
knrl r = 0.81 r = 0.923
Kr r = 0.757 r = 0.926
lok r = 0.68 r = 0.909
Mdr49 r = 0.816 r = 0.939
Mes2 r = 0.878 r = 0.928
MESR3 r = 0.757 r = 0.915
mfas r = 0.444 r = 0.769
Nek2 r = 0.521 r = 0.84
NetA r = 0.705 r = 0.896
noc r = 0.577 r = 0.866
nub r = 0.816 r = 0.931
numb r = 0.483 r = 0.889
oc r = 0.859 r = 0.877
odd r = 0.681 r = 0.83
peb r = 0.353 r = 0.586
prd r = 0.676 r = 0.869
pxb r = 0.597 r = 0.838
rau r = 0.606 r = 0.869
rho r = 0.79 r = 0.891
run r = 0.658 r = 0.894
sna r = 0.934 r = 0.94
srp r = 0.606 r = 0.753
tkv r = 0.28 r = 0.565
tll r = 0.462 r = 0.611
toc r = 0.688 r = 0.907
Traf4 r = 0.594 r = 0.87
trn r = 0.712 r = 0.929
tsh r = 0.642 r = 0.831
twi r = 0.936 r = 0.937
zen r = 0.829 r = 0.817
zen2 r = 0.386 r = 0.614
zfh1 r = 0.215 r = 0.442

Supplementary Figure 5: Improved correlation via hyperparameter optimization

Improved correlation between predicted and referenced data in the scRNA-seq space by optimizing the weighting function for all landmark genes. Parameters of Perler are listed in Supplementary Table 7.

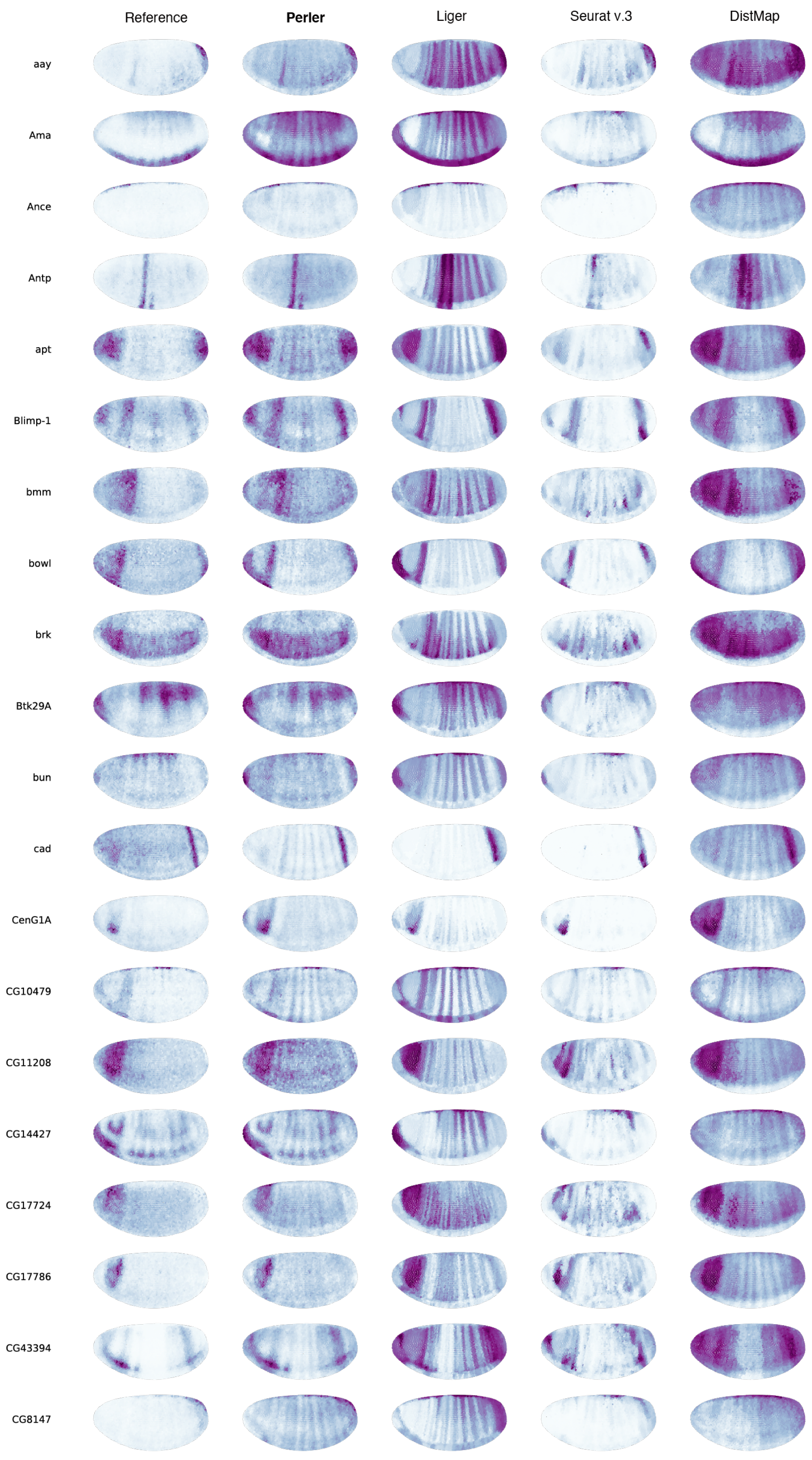

Supplementary Figure 6: Spatial reconstruction of all landmark genes

Spatial reconstruction of all landmark genes (84 genes) by Perler, Liger, Seurat (v.3), and DistMap. Parameters of Perler are listed in Supplementary Table 7. This supplementary figure continues the following 4 pages.

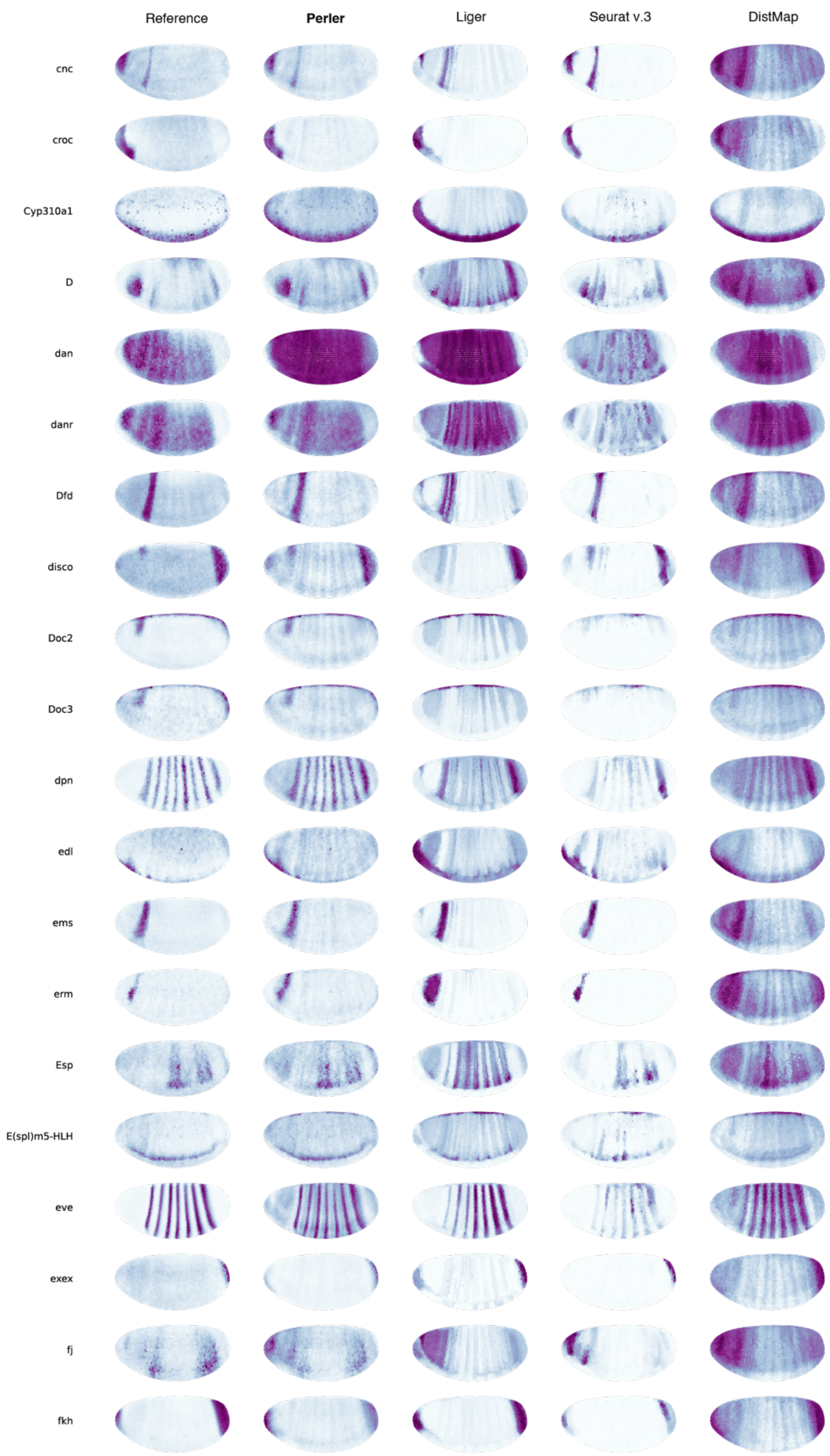

Supplementary Figure 6 (2)

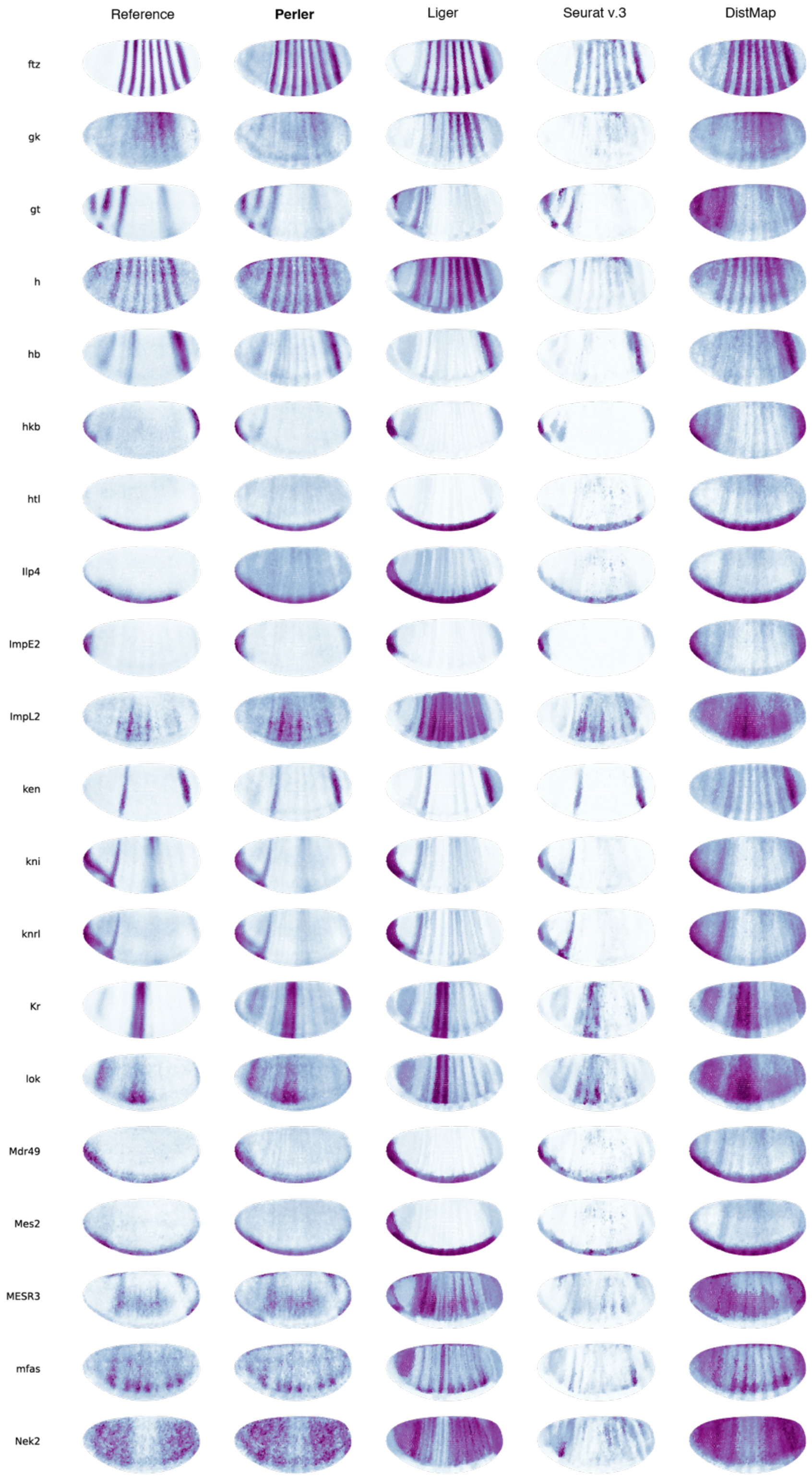

Supplementary Figure 6 (3)

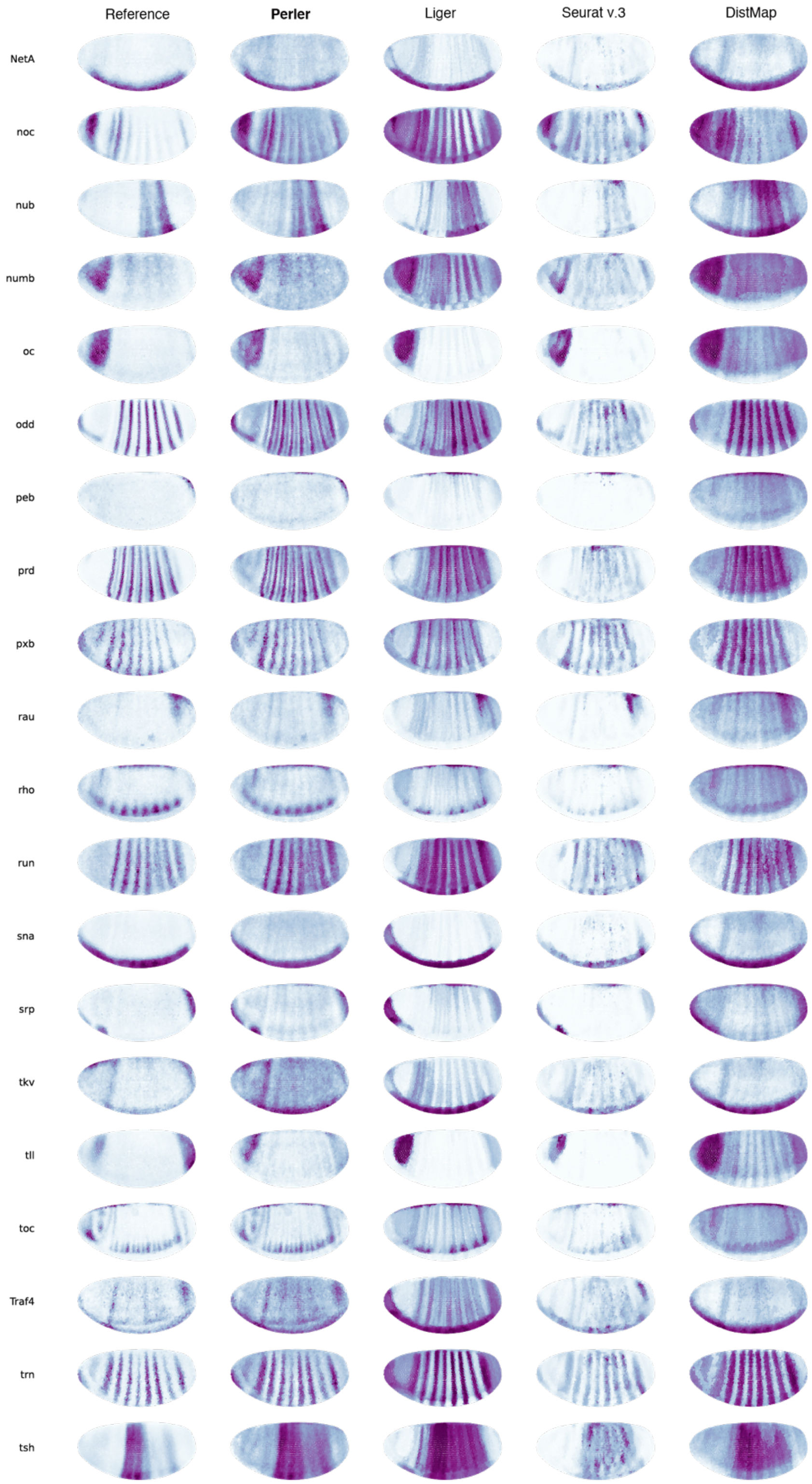

Supplementary Figure 6 (4)

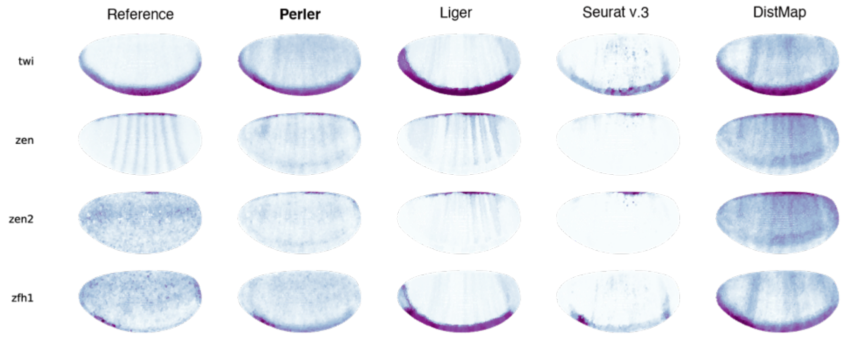

Supplementary Figure 6 (5)

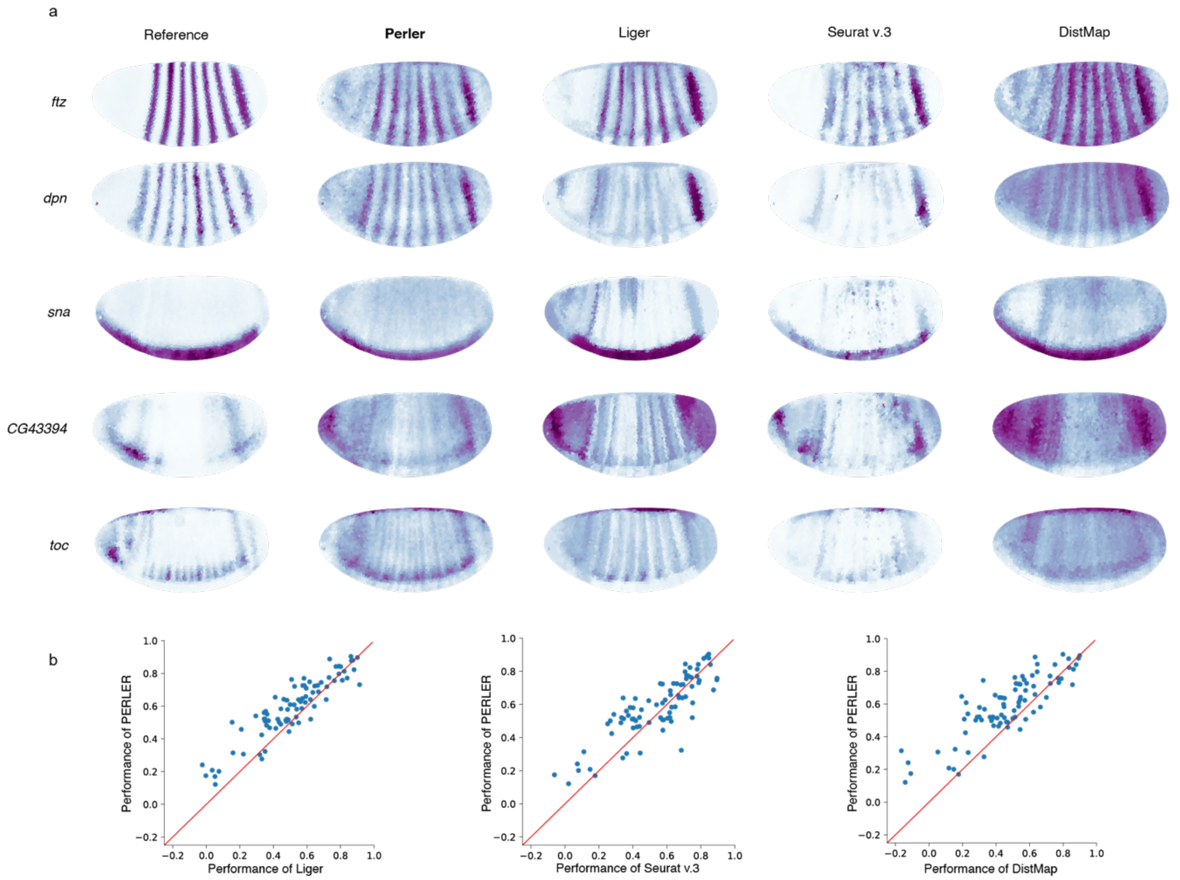

Supplementary Figure 7: Spatial prediction of landmark genes

(a) Predictions of landmark gene expression by Perler. Left and right panels depict the spatial reference maps and the predicted spatial gene-expression profiles. For each prediction, the predicted gene was removed from the reference ISH data (LOOCV). (b) Performance comparison of Perler with Liger (left, two-sided Wilcoxon test: $p = 2.3 \times 10^{-9}$), Seurat (v.3) (middle, two-sided Wilcoxon test: $p = 3.4 \times 10^{-3}$), and DistMap (right, two-sided Wilcoxon test: $p = 6.6 \times 10^{-11}$). Each dot indicates the predictive accuracies for each gene by Perler and previous methods. Red lines depict auxiliary lines showing the same performance of two methods. Parameters of Perler are listed in Supplementary Table 7.

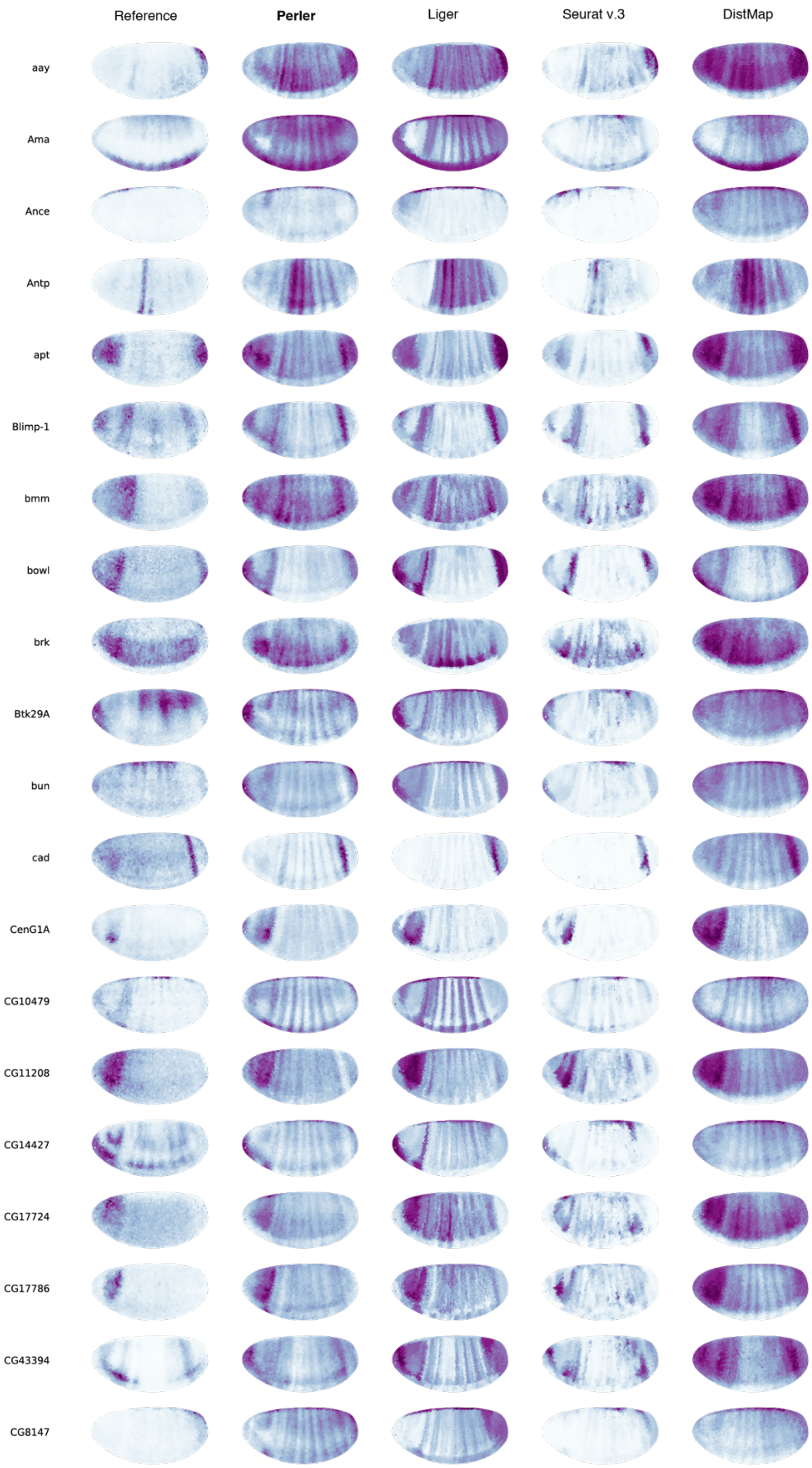

Supplementary Figure 8: Spatial prediction of all landmark genes
Spatial prediction of all landmark genes (84 genes) by Perler, Liger, Seurat (v.3), and DistMap. The spatial prediction was generated by LOOCV experiments. Parameters of Perler are listed in Supplementary Table 7. This supplementary figure continues the following 4 pages.

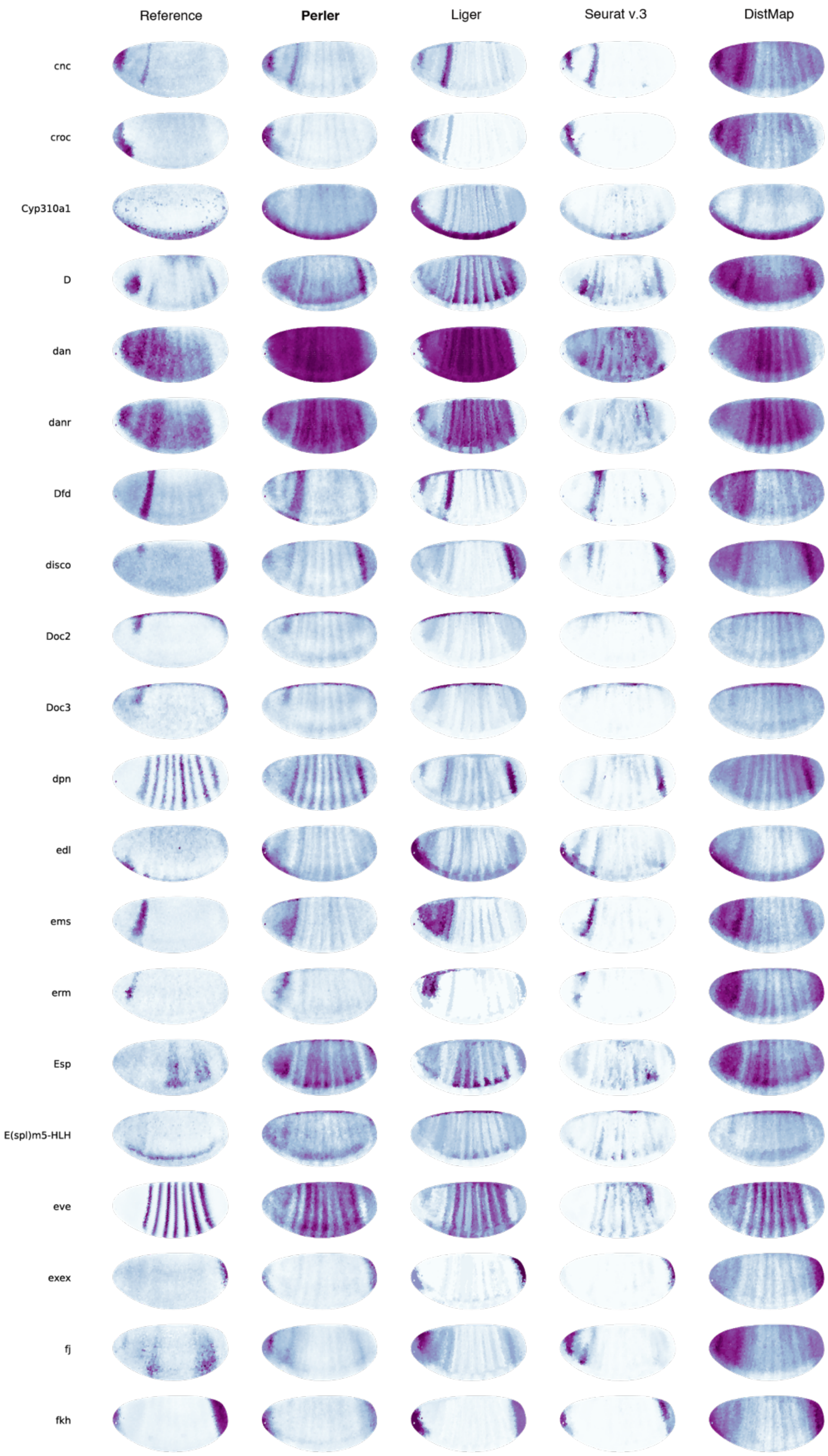

Supplementary Figure 8 (2)

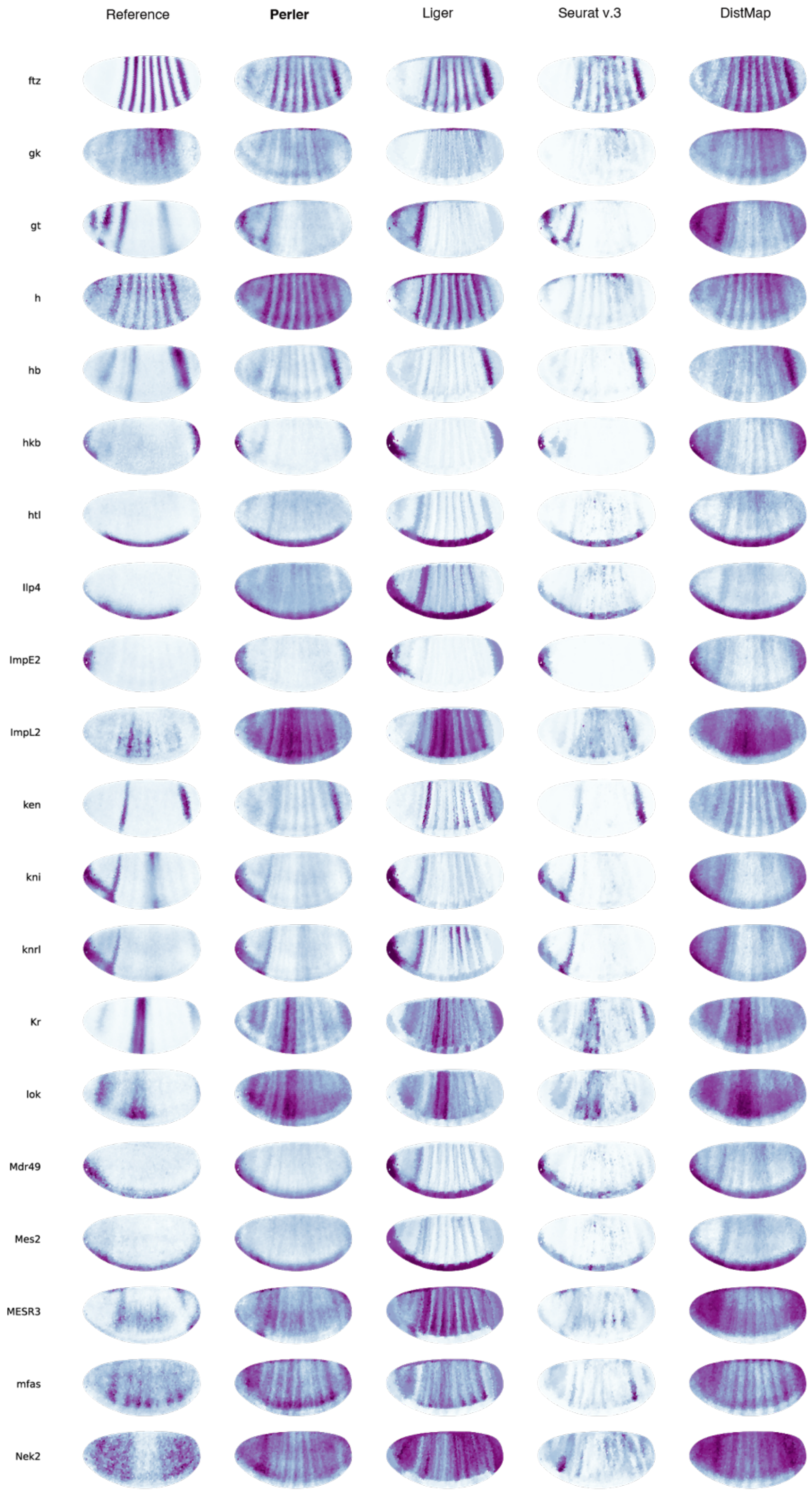

Supplementary Figure 8 (3)

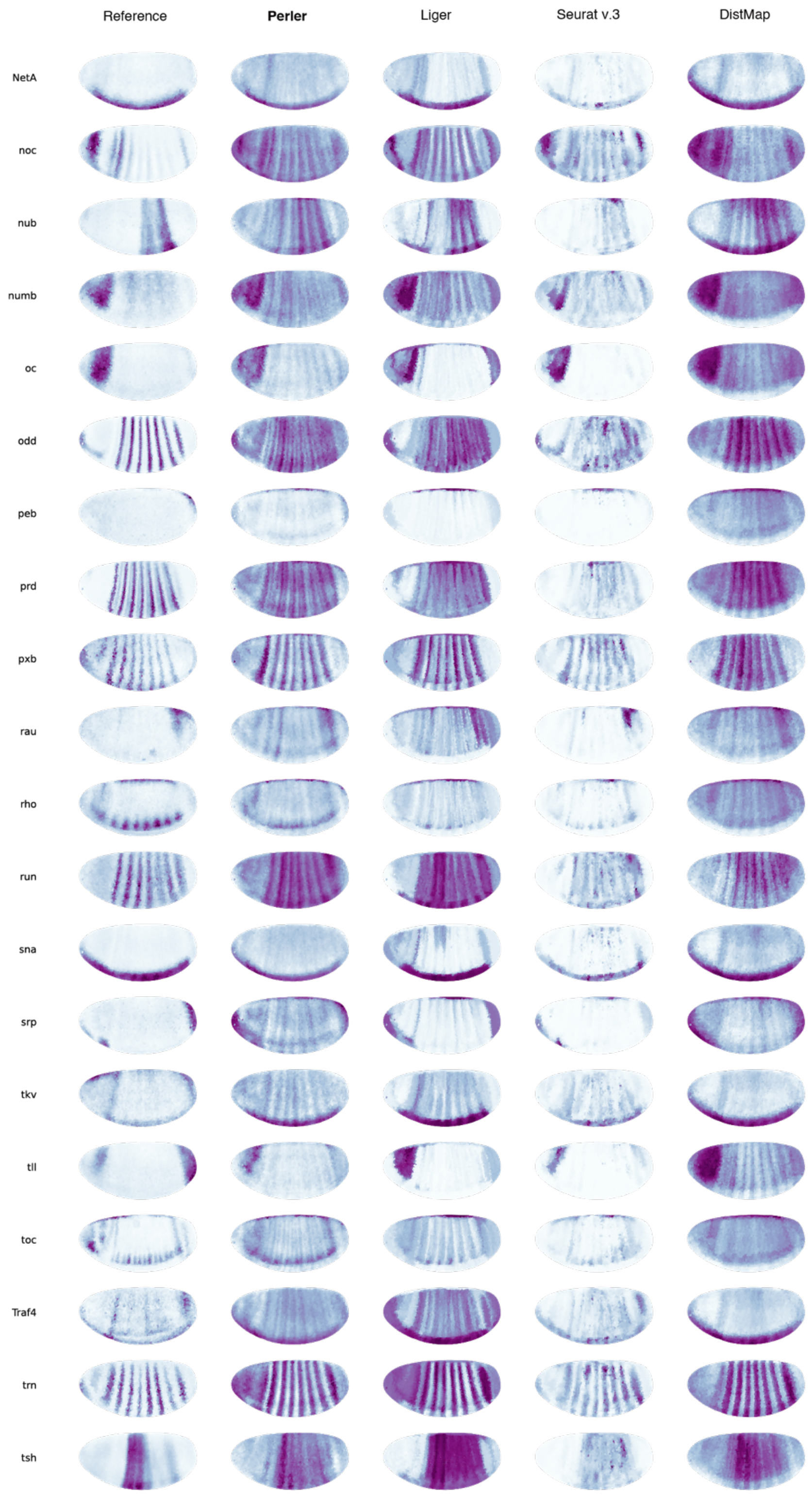

Supplementary Figure 8 (4)

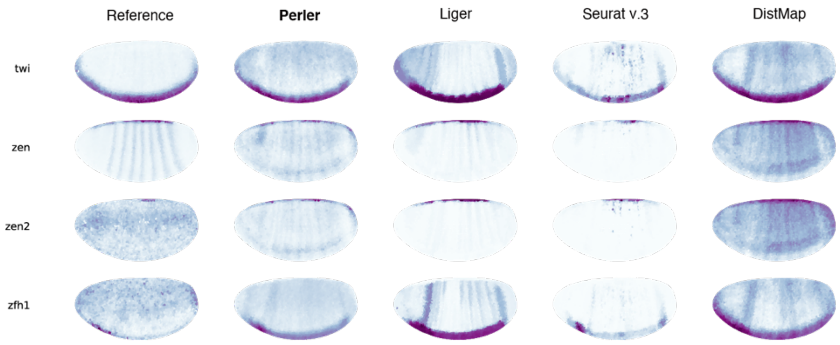

Supplementary Figure 8 (5)

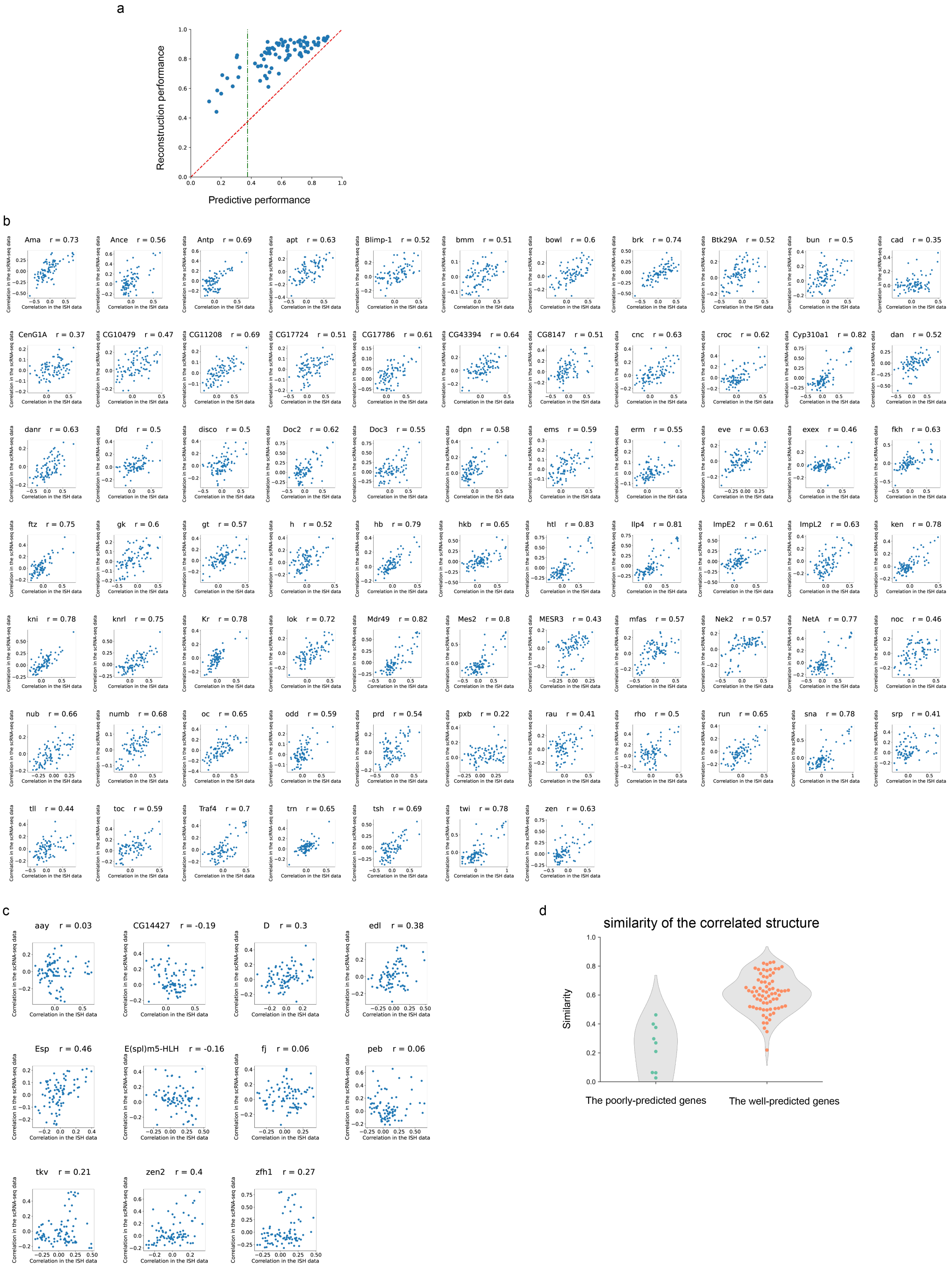
a
Reconstruction performance
Predictive performance
b
Ama r = 0.73
Ance r = 0.56
Antp r = 0.69
apt r = 0.63
Blimp-1 r = 0.52
bmm r = 0.51
bowl r = 0.6
brk r = 0.74
Btk29A r = 0.52
bun r = 0.5
cad r = 0.35
CenG1A r = 0.37
CG10479 r = 0.47
CG11208 r = 0.69
CG17724 r = 0.51
CG17786 r = 0.61
CG43394 r = 0.64
CG8147 r = 0.51
cnc r = 0.63
croc r = 0.62
Cyp310a1 r = 0.82
dan r = 0.52
danr r = 0.63
Dfd r = 0.5
disco r = 0.5
Doc2 r = 0.62
Doc3 r = 0.55
dpn r = 0.58
ems r = 0.59
erm r = 0.55
eve r = 0.63
exex r = 0.46
fkh r = 0.63
ftz r = 0.75
gk r = 0.6
gt r = 0.57
h r = 0.52
hb r = 0.79
hkb r = 0.65
htl r = 0.83
Ilp4 r = 0.81
ImpE2 r = 0.61
ImpL2 r = 0.63
ken r = 0.78
kni r = 0.78
knrl r = 0.75
Kr r = 0.78
lok r = 0.72
Mdr49 r = 0.82
Mes2 r = 0.8
MESR3 r = 0.43
mfas r = 0.57
Nek2 r = 0.57
NetA r = 0.77
noc r = 0.46
nub r = 0.66
numb r = 0.68
oc r = 0.65
odd r = 0.59
prd r = 0.54
pxb r = 0.22
rau r = 0.41
rho r = 0.5
run r = 0.65
sna r = 0.78
srp r = 0.41
tll r = 0.44
toc r = 0.59
Traf4 r = 0.7
trn r = 0.65
tsh r = 0.69
twi r = 0.78
zen r = 0.63
Correlation in the scRNA-seq data
Correlation in the ISH data
c
aay r = 0.03
CG14427 r = -0.19
D r = 0.3
edl r = 0.38
Esp r = 0.46
E(spl)m5-HLH r = -0.16
fj r = 0.06
peb r = 0.06
tkv r = 0.21
zen2 r = 0.4
zfh1 r = 0.27
Correlation in the scRNA-seq data
Correlation in the ISH data
d
similarity of the correlated structure
Similarity
The poorly-predicted genes
The well-predicted genes

Supplementary Figure 9: The well-predicted and poorly-predicted genes

(a) Comparison between Perler's reconstruction accuracy and its predictive accuracy for *Drosophila* data. Each dot indicates the reconstruction/prediction accuracy of each gene by Perler. The green dashed line indicates the criterion used to classify landmark genes as well- or poorly-predicted genes. Red lines depict auxiliary lines showing the same reconstruction and prediction performance. Parameters of Perler are listed in Supplementary Table 7. (b, c) Each dot indicates the relationship between 'correlation with all landmark genes in ISH dataset' and 'correlation with all landmark genes in scRNA-seq dataset' for each well-predicted gene (b) and each poorly-predicted gene (c). Correlation coefficients for these scatter plots were evaluated as the similarities in the gene expression pattern between the ISH and the scRNA-seq data for each gene. (d) Each dot indicates the correlation coefficient for the scatter plots in (b) and (c), which represent the similarity of the gene expression pattern between the ISH and scRNA-seq data for each gene based on the correlated data structures among all genes.

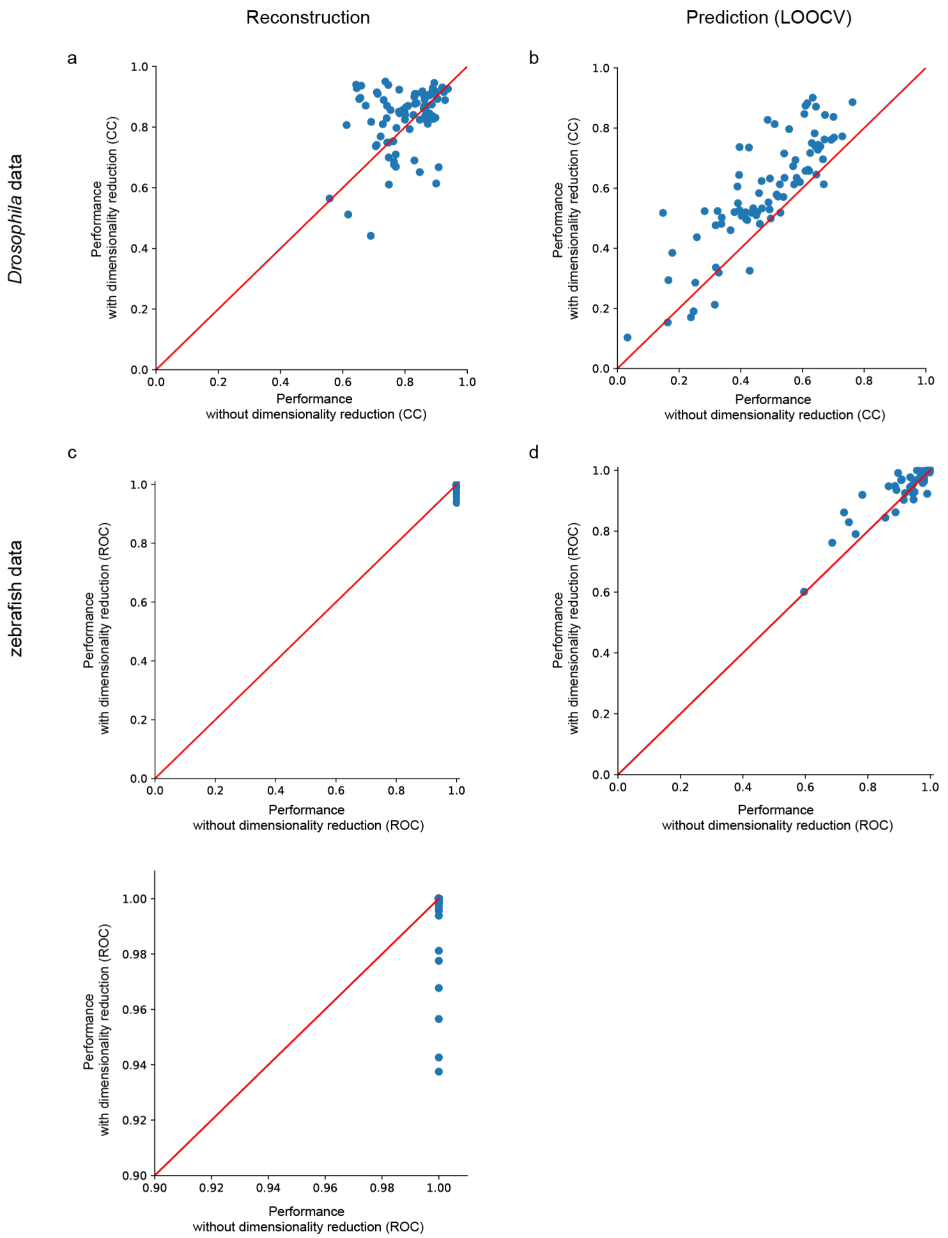

Supplementary Figure 10: Performance improvement by dimensionality reduction
Comparison between Perler's performance with and without dimensionality reduction. (a) Comparison of the reconstruction accuracy in the *Drosophila* data. Average correlation coefficient (aCC) is 0.83 and 0.79 for the performance with and without dimensionality reduction, respectively. (b) Comparison of the predictive accuracy in the *Drosophila* data. aCC is 0.59 and 0.48 for the performance with and without dimensionality reduction, respectively. (c) Comparison of the reconstruction accuracy in the zebrafish data. Median ROC score is 1.0 and 1.0 for the performance with and without dimensionality reduction, respectively. Note, the lower panel indicates the enlarged panel of the upper panel. (d) Comparison of the predictive accuracy in the zebrafish data. Median ROC score is 0.97 and 0.96 for the performance with and without dimensionality reduction, respectively. Each dot indicates the reconstruction/prediction accuracy for each landmark gene by Perler. Note, we did not conduct this type of experiment using the mammalian liver data or mouse cortex data because the mammalian liver data has too few landmark genes (6 genes) to utilize for dimensionality reduction, while the mouse cortex data has too many landmark genes (1,080 genes) to examine Perler's performance without dimensionality reduction. Red lines depict auxiliary lines showing the same performance with and without dimensionality reduction. Parameters of Perler are listed in Supplementary Table 7.

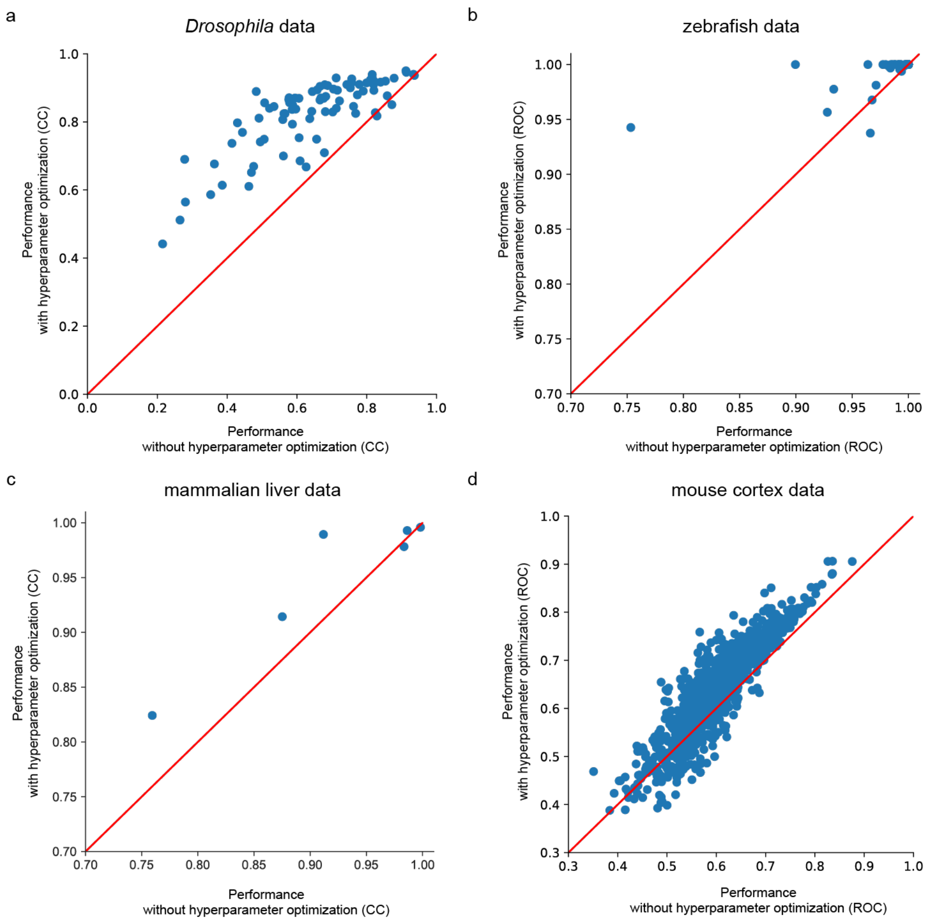

Supplementary Figure 11: Performance improvement via hyperparameter optimization
Comparison between Perler's performance with and without hyperparameter optimization. (a) Comparison in the *Drosophila* data. Average correlation coefficient (aCC) is 0.83 and 0.65 for the performance with and without hyperparameter optimization, respectively. (b) Comparison in the zebrafish data. Median ROC score is 1.0 and 1.0 for the performance with and without hyperparameter optimization, respectively. (c) Comparison in the mammalian liver data. Average correlation coefficient (aCC) is 0.95 and 0.92 for the performance with and without hyperparameter optimization. (d) Comparison in the mouse cortex data. Each dot indicates the reconstruction accuracy for each gene by Perler. Median ROC score is 0.65 and 0.60 for the performance with and without hyperparameter optimization, respectively. Red lines depict auxiliary lines showing the same performance with and without hyperparameter optimization. Parameters of Perler are listed in Supplementary Table 7.

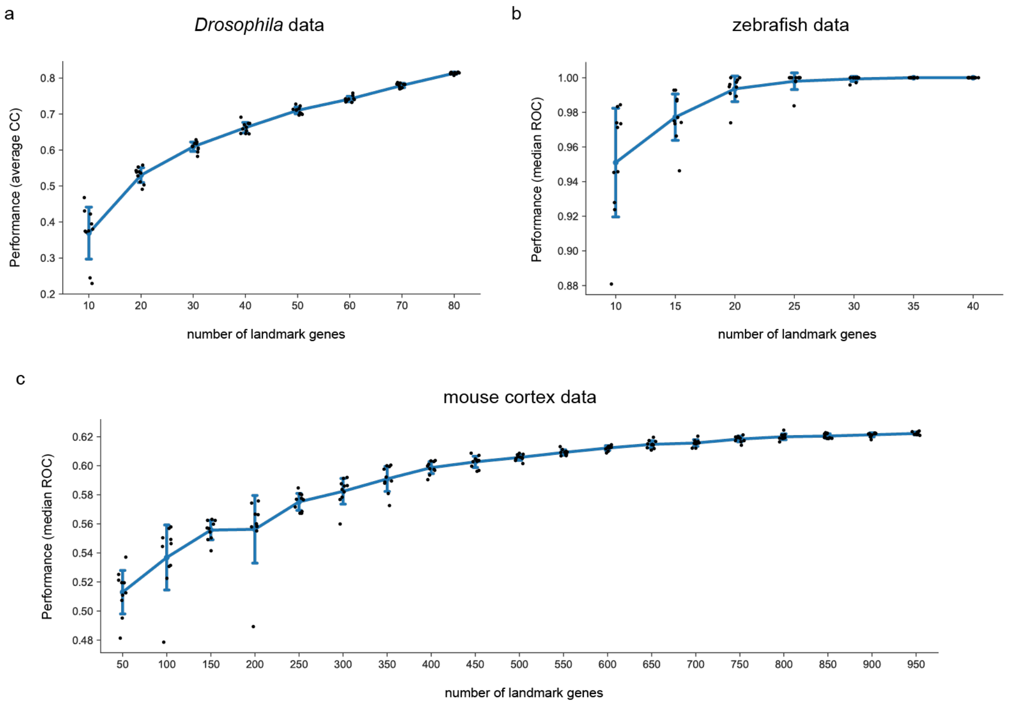

Supplementary Figure 12: Performance depending on the number of landmark genes

Perler's reconstruction performance was examined by randomly down-sampling different numbers of landmark genes for the *Drosophila* data (a), zebrafish data (b), and mouse cortex data (c). Each blue line indicates mean of all ten trials. Each error bar represents standard deviation. Parameters of Perler are listed in Supplementary Table 7.

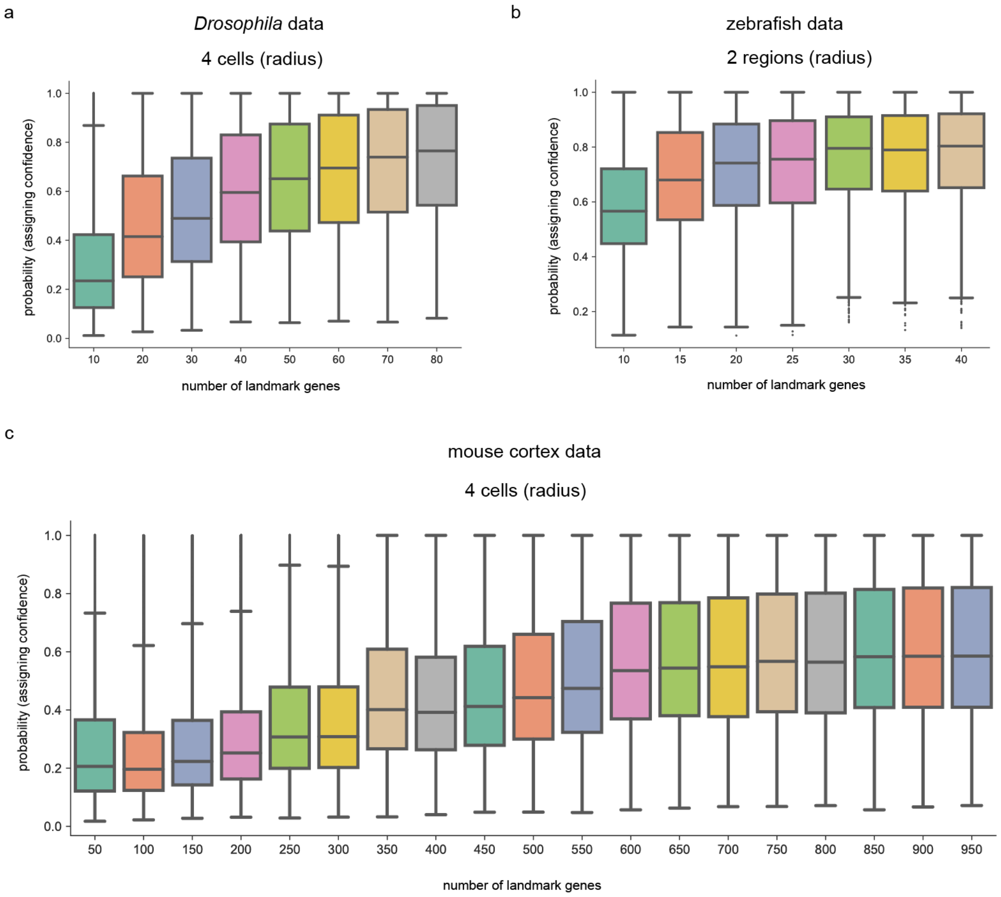

Supplementary Figure 13: Assigned specificity depending on the number of landmark genes
The assigned specificity (related to Figure 2d) was examined by randomly down-sampling different numbers of landmark genes for the *Drosophila* data (a), zebrafish data (b), and mouse cortex data (c). The assigned specificity was calculated by the posterior probabilities of circular regions for each scRNA-seq data point according to radius, with the center of each region representing the optimally assigned location for each data point. The radius was calculated by path length on the k-NN graph comprising all cells in the tissue. For the box signifies the upper and lower quartiles, and the median is represented by a short black line within the box. The whiskers in the boxplot have a maximum 1.5 interquartile range, with black points indicating outliers. n=1297 (a), 851 (b), and 14249 (c) biologically independent cells (scRNA-seq data points). Parameters of Perler are listed in Supplementary Table 7.

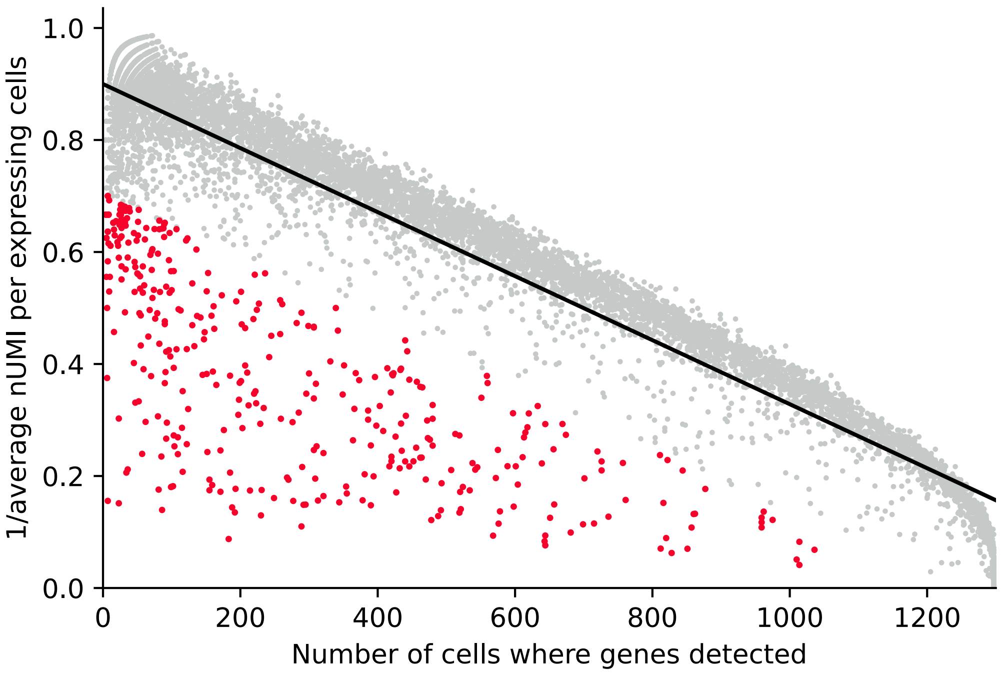

Supplementary Figure 14: Identification of spatially restricted genes (SRGs)

Scatter plot identifying SRGs. The red and grey points indicate SRGs and other genes, respectively. The black line indicates the linear regression. The region of SRGs was defined by the area under the minus-2 standard deviations of the regression line.

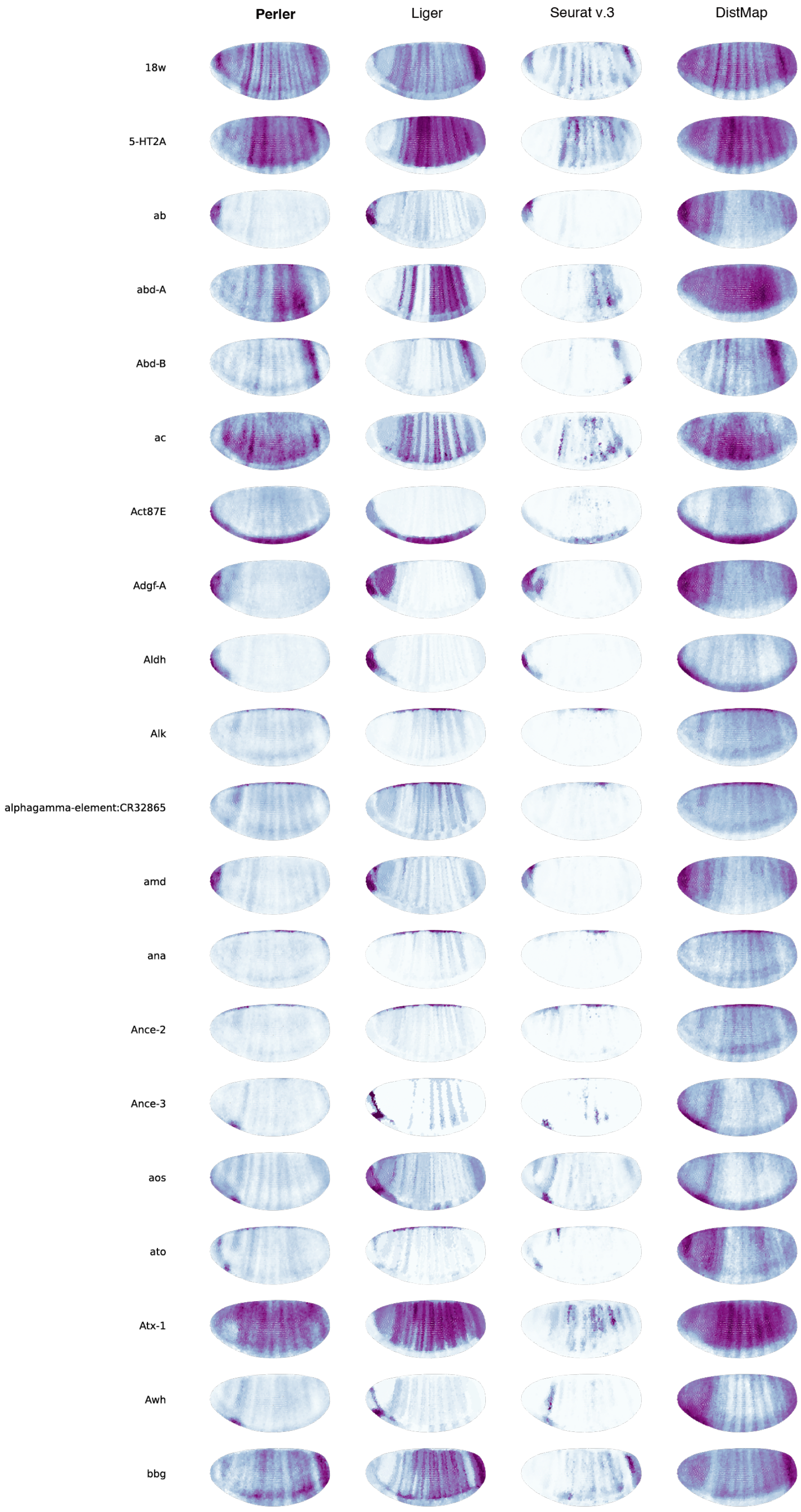

Supplementary Figure 15: Spatial prediction of non-landmark spatially restricted genes (SRGs)
Predictions of expression of non-landmark SRGs (310 genes) were selected in Supplementary Fig. 14 by Perler, Liger, Seurat (v.3), and DistMap. Parameters of Perler are listed in Supplementary Table 7. This supplementary figure continues the following 15 pages.

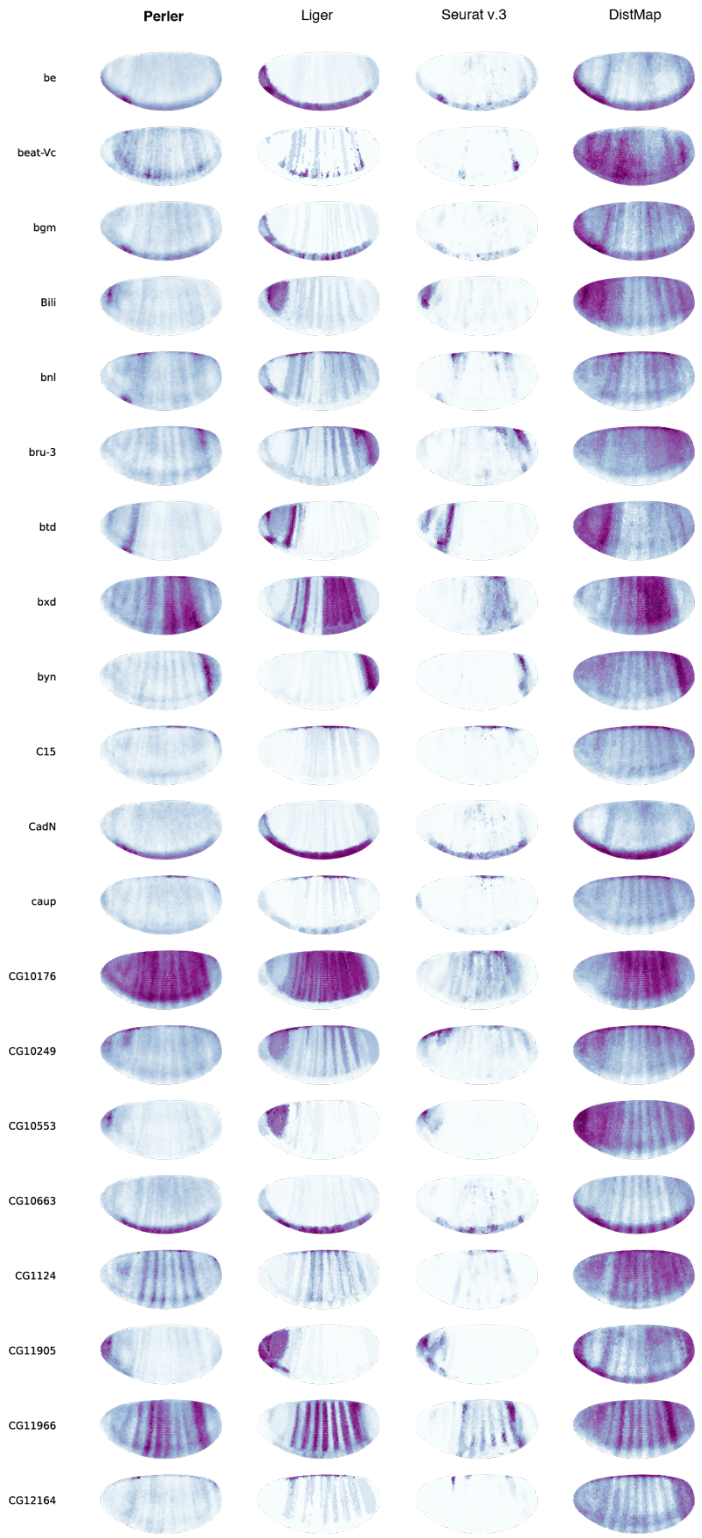

Supplementary Figure 15 (2)

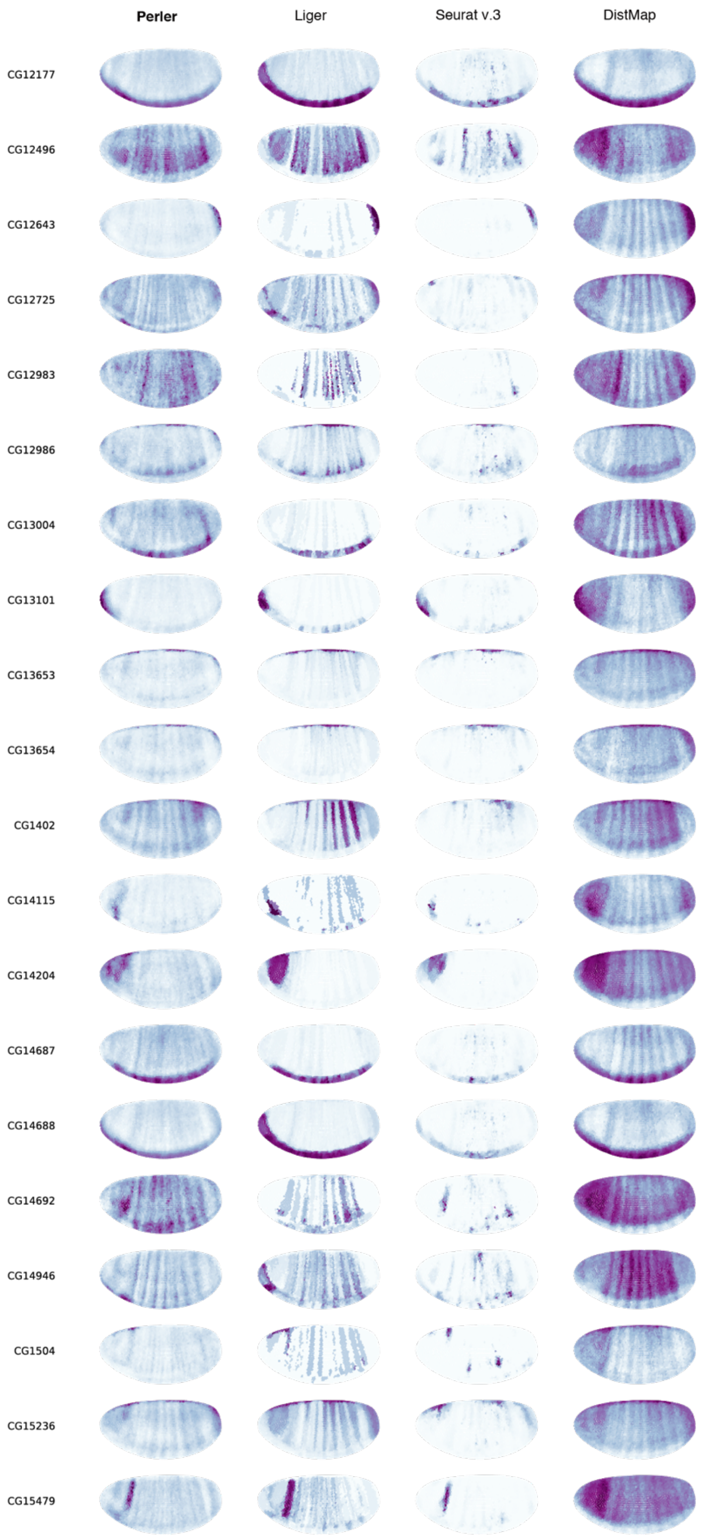

Supplementary Figure 15 (3)

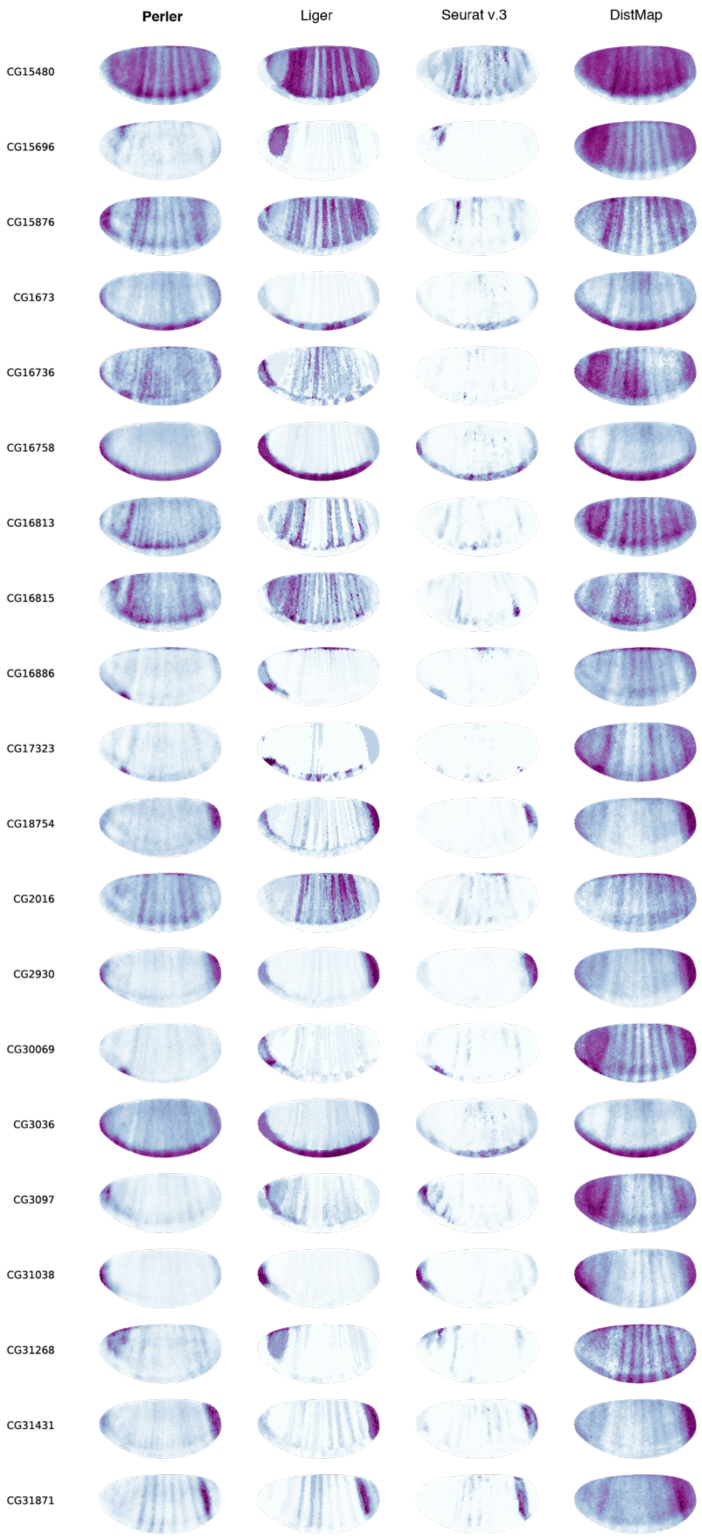

Supplementary Figure 15 (4)

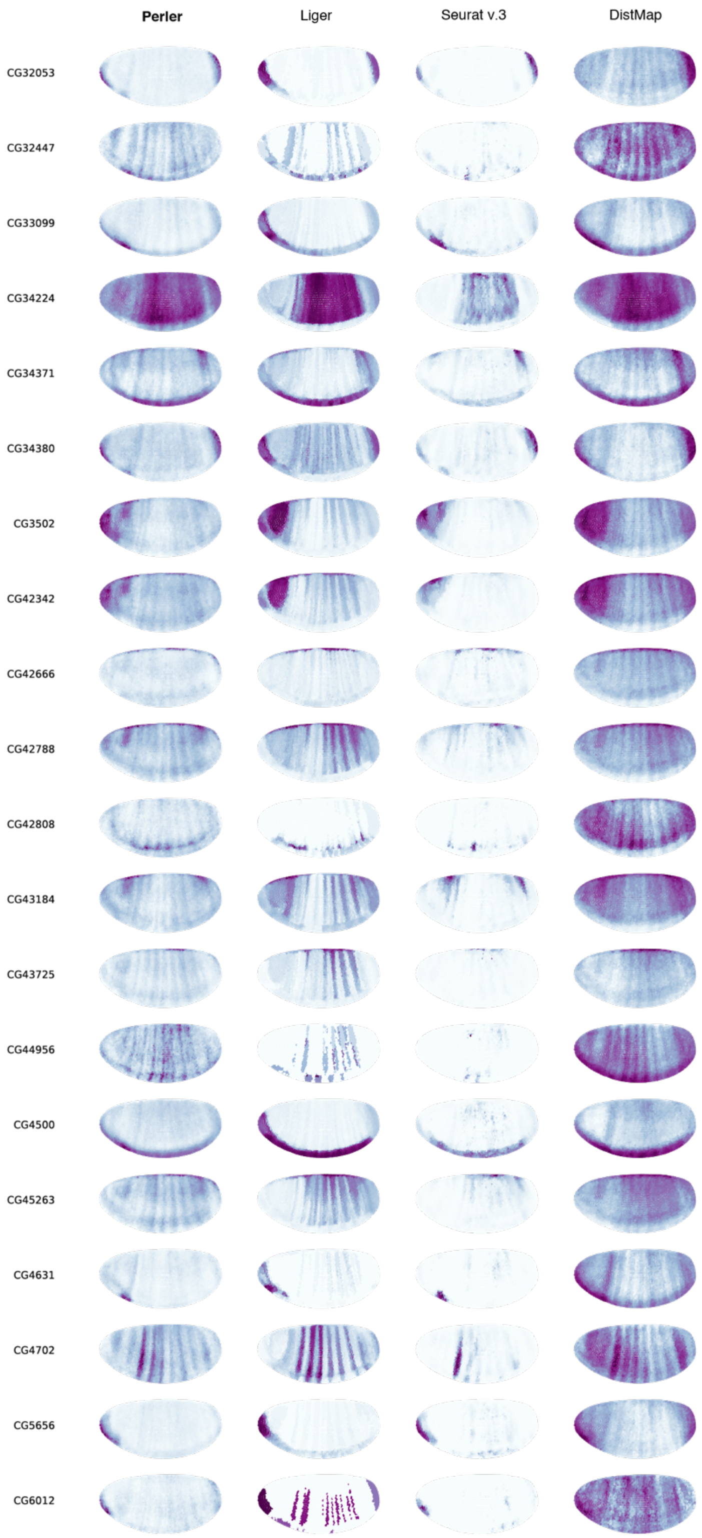

Supplementary Figure 15 (5)

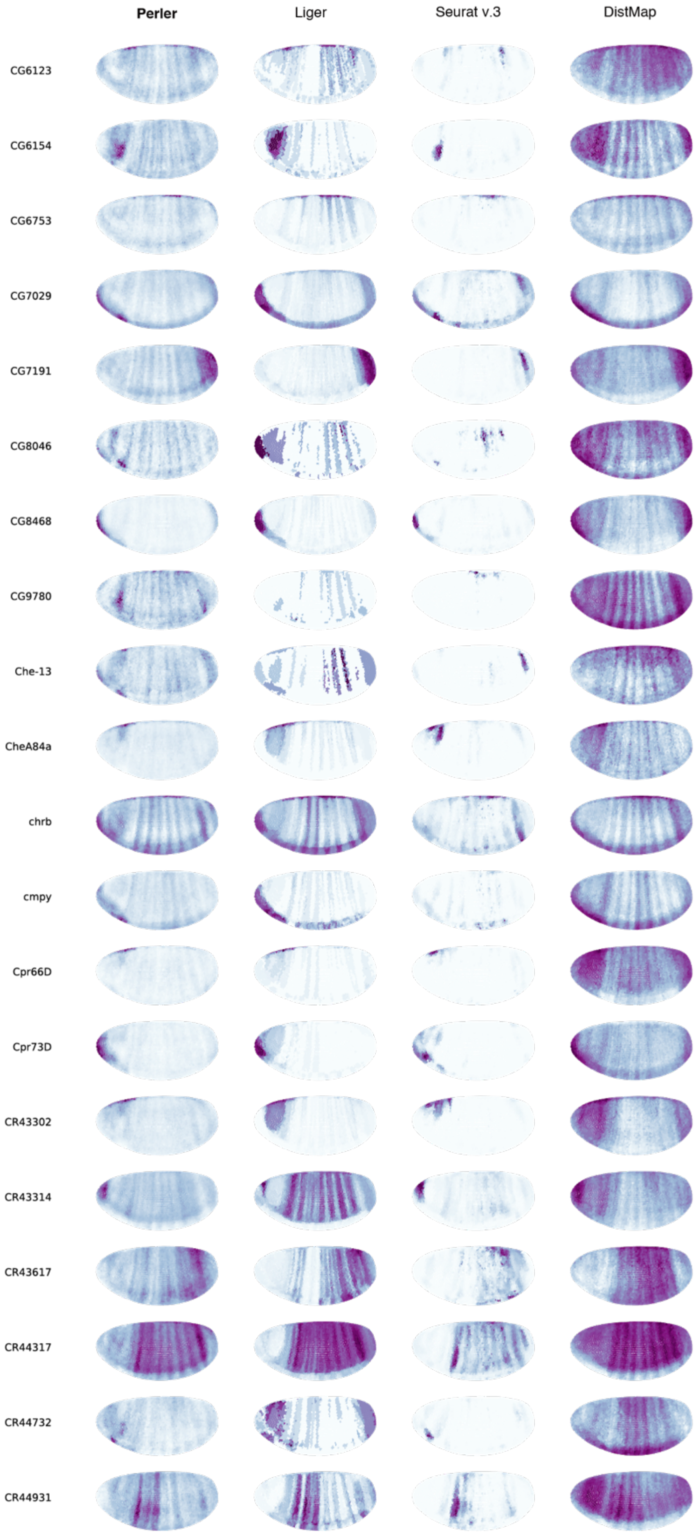

Supplementary Figure 15 (6)

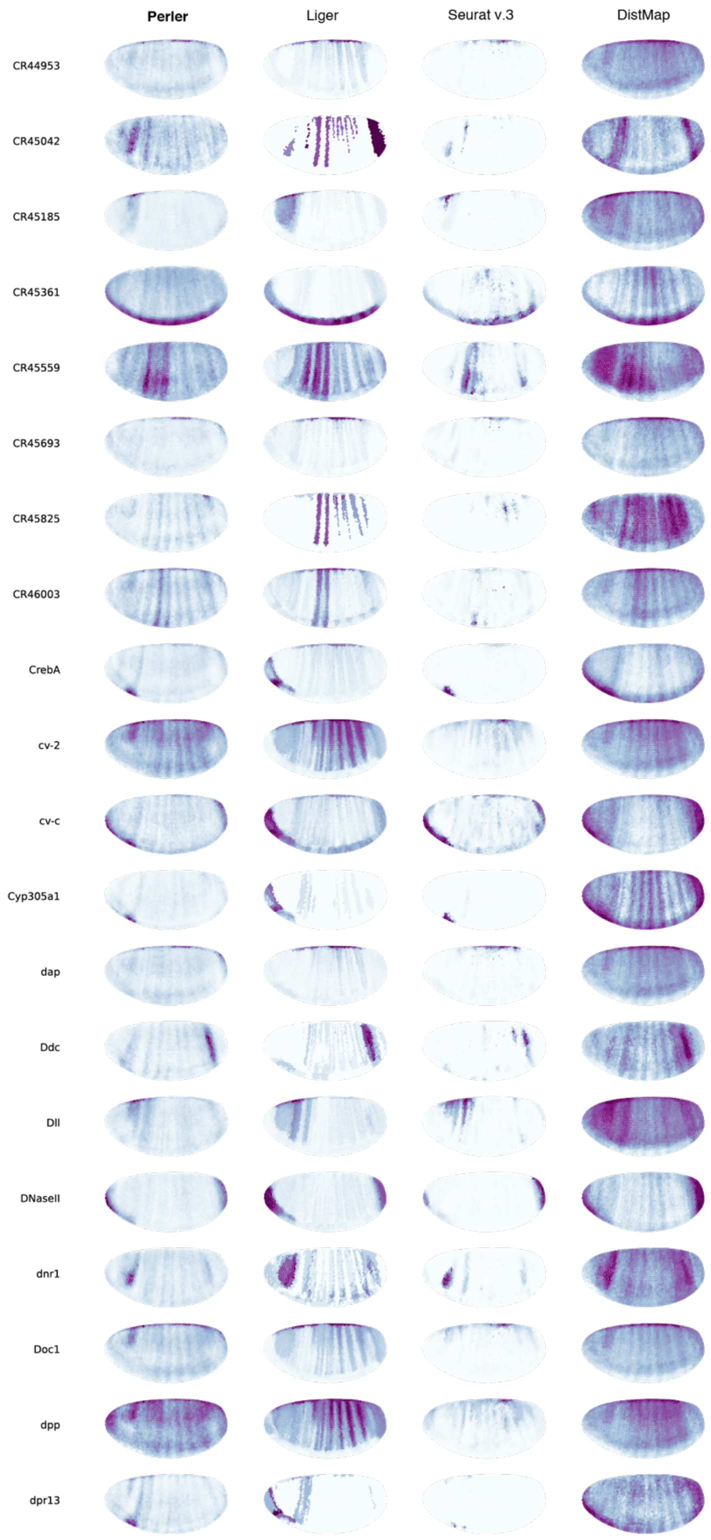

Supplementary Figure 15 (7)

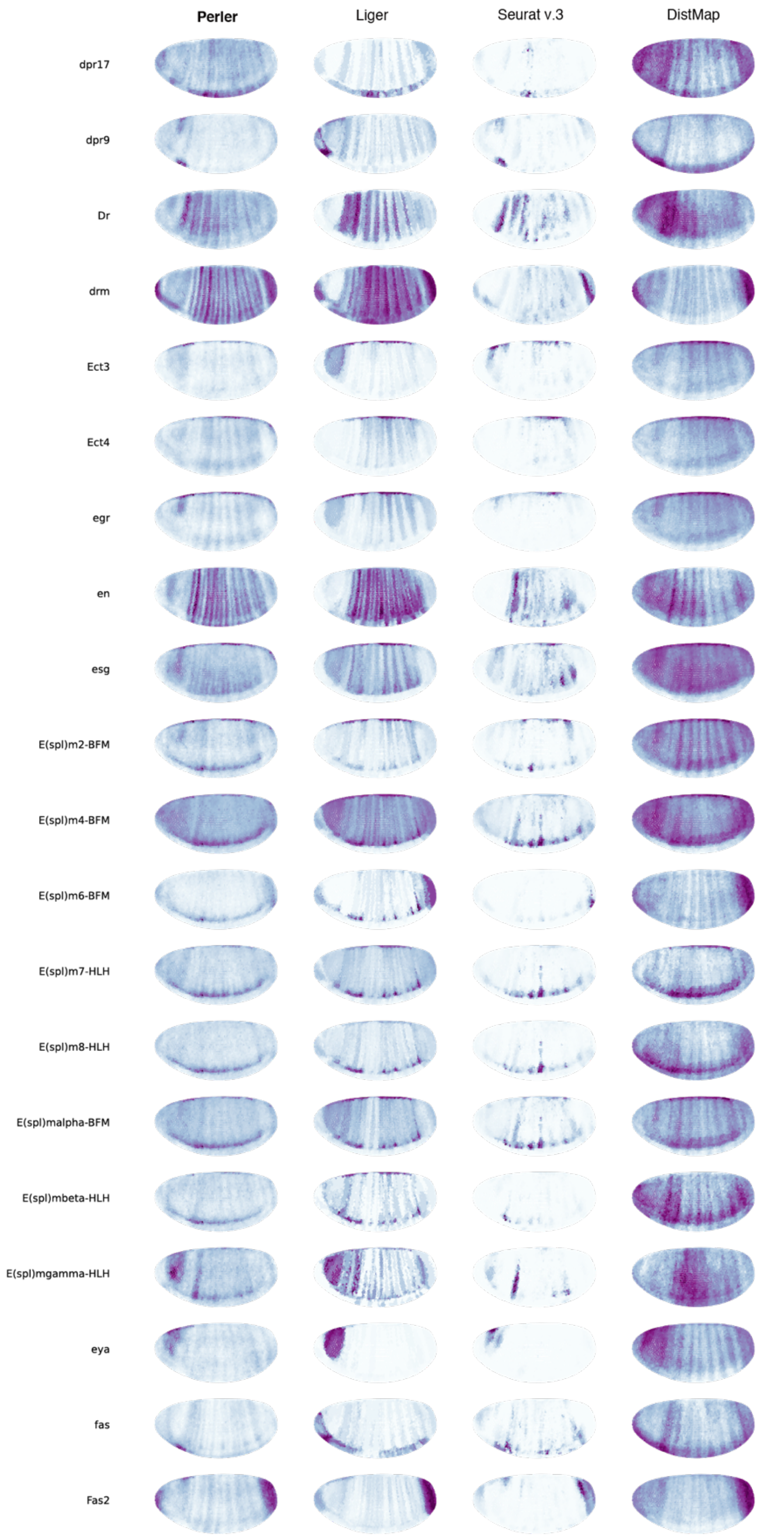

Supplementary Figure 15 (8)

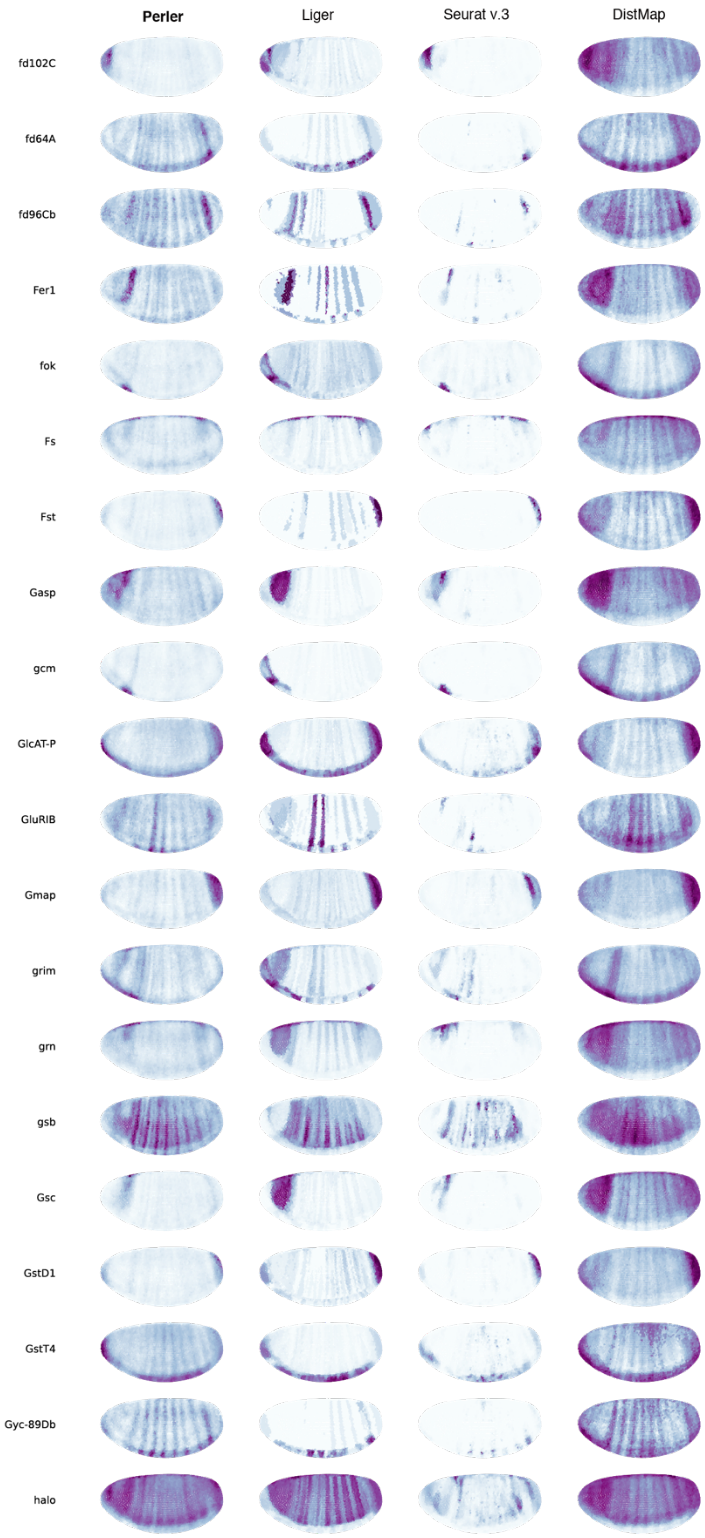

Supplementary Figure 15 (9)

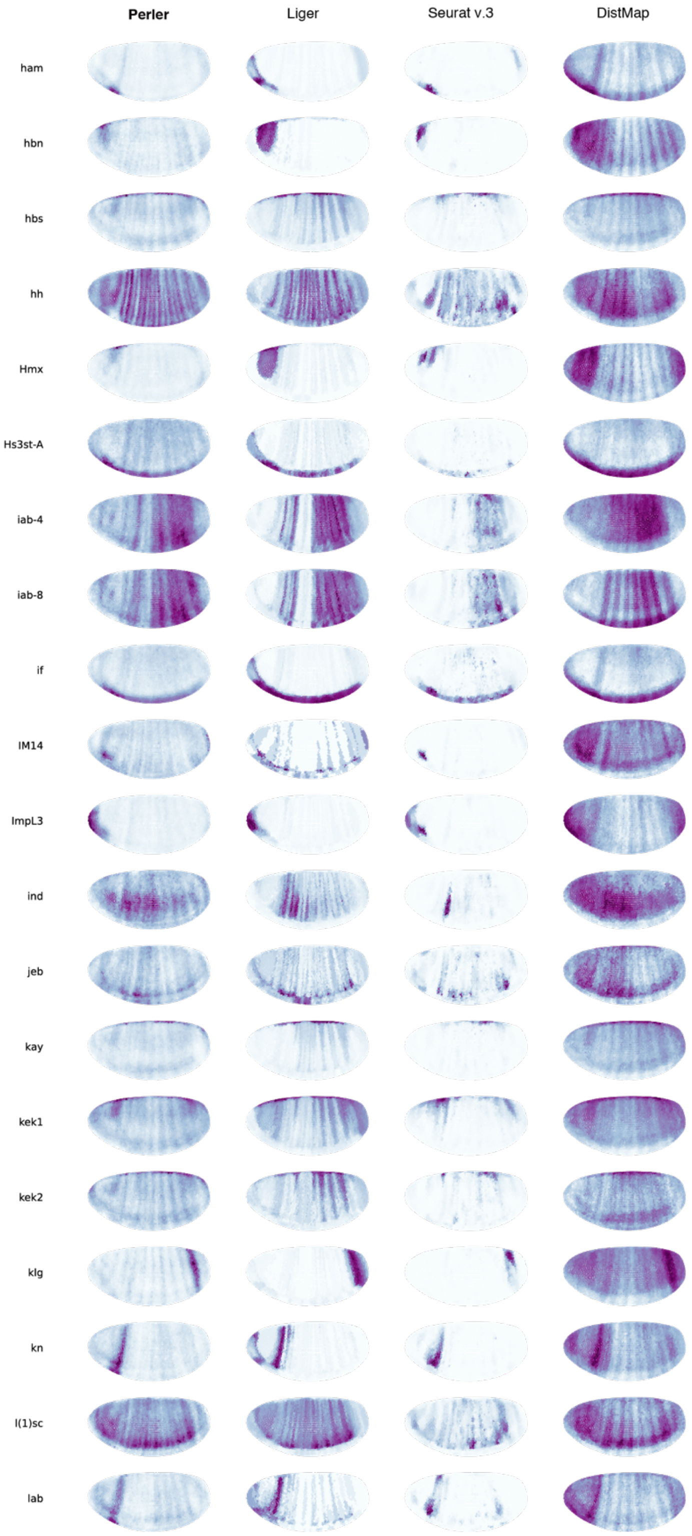

Supplementary Figure 15 (10)

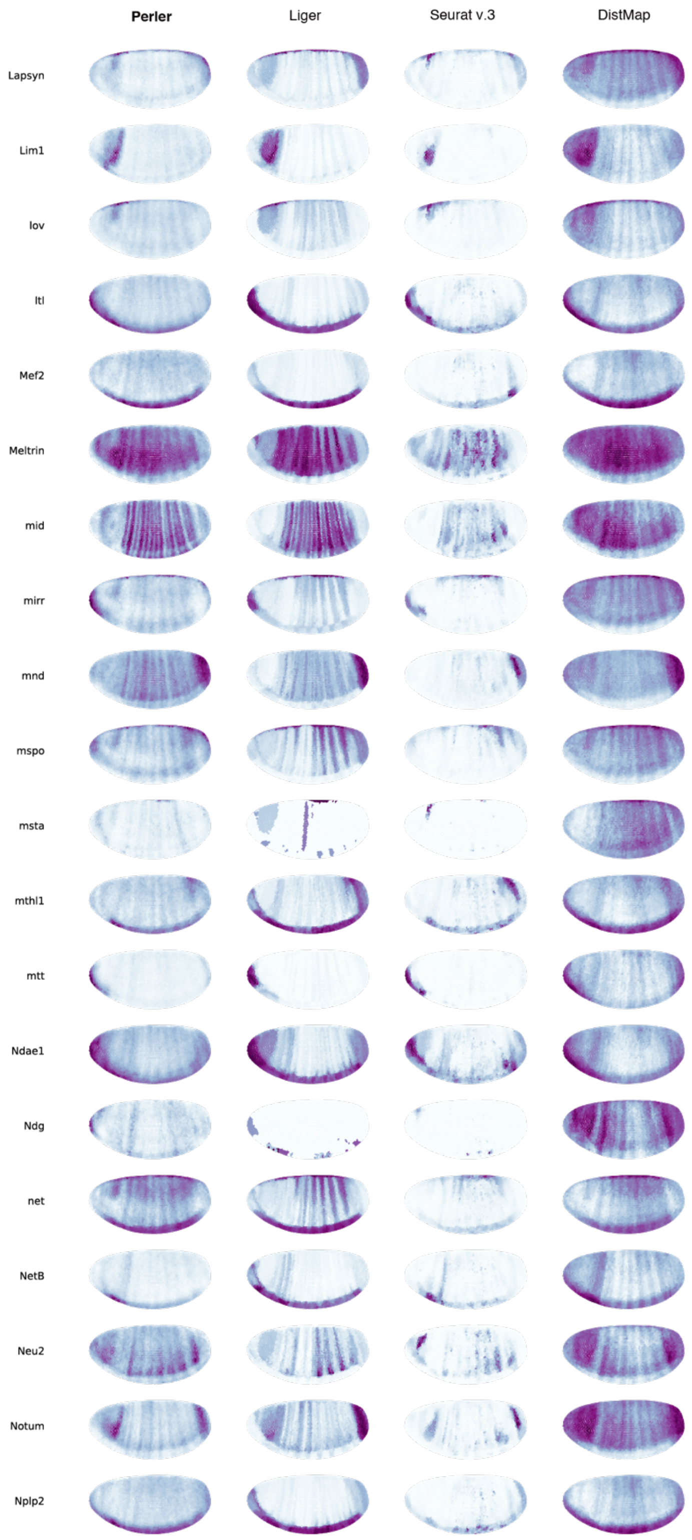

Supplementary Figure 15 (11)

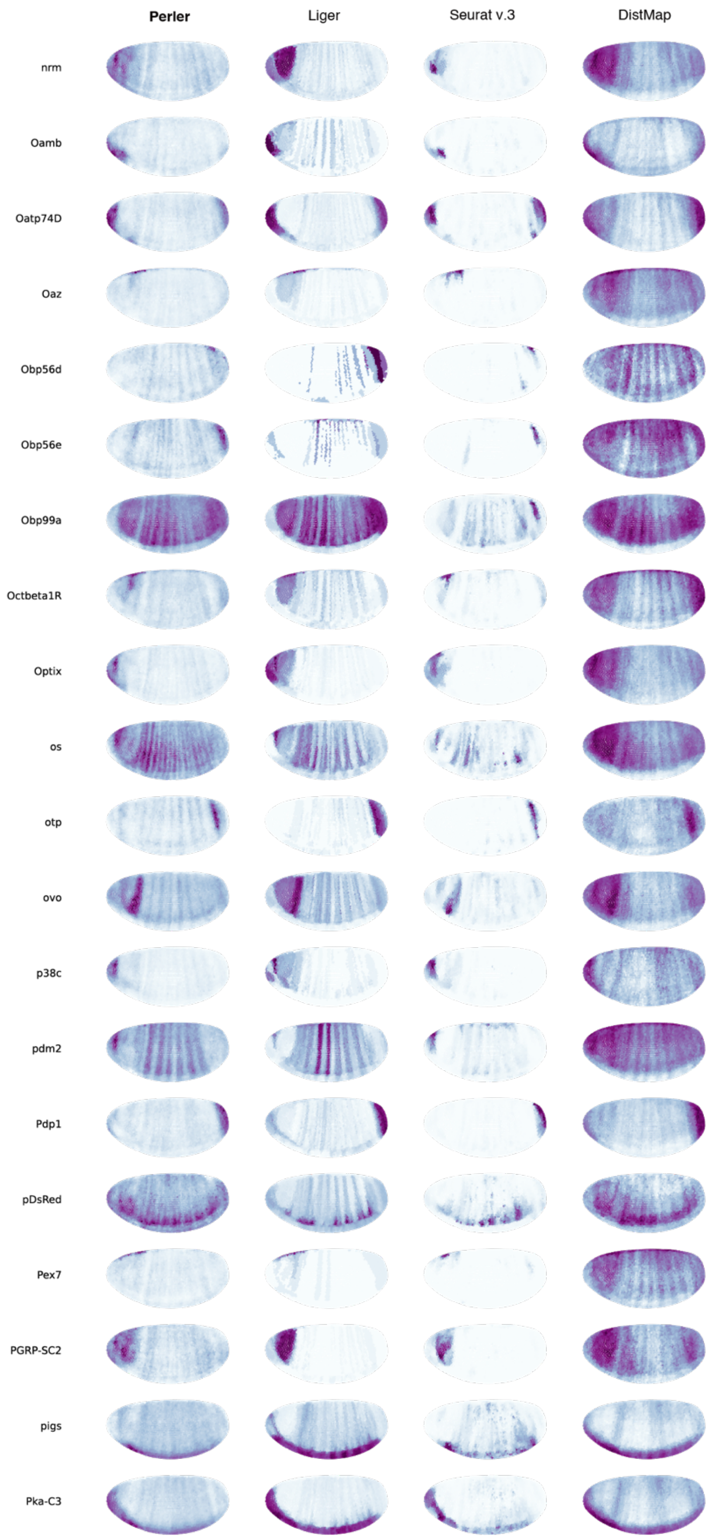

Supplementary Figure 15 (12)

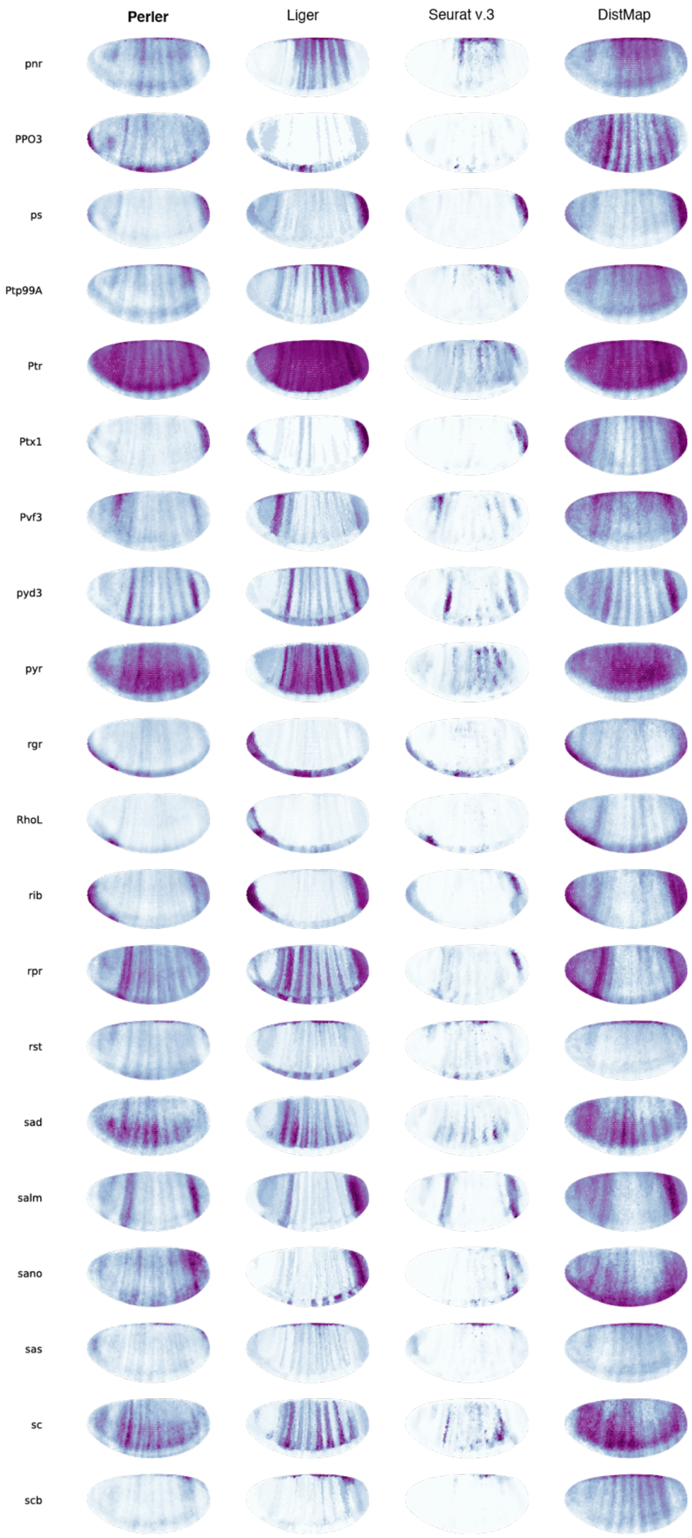

Supplementary Figure 15 (13)

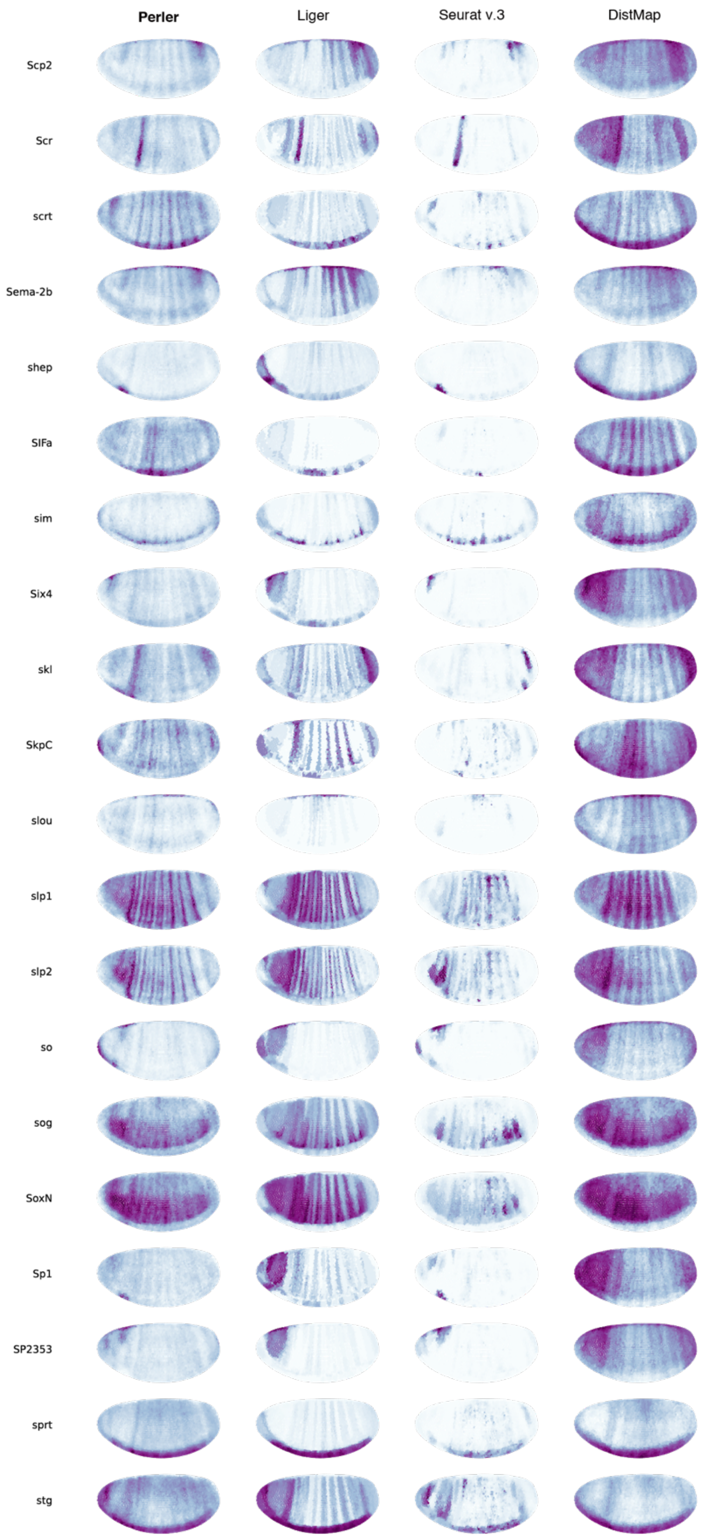

Supplementary Figure 15 (14)

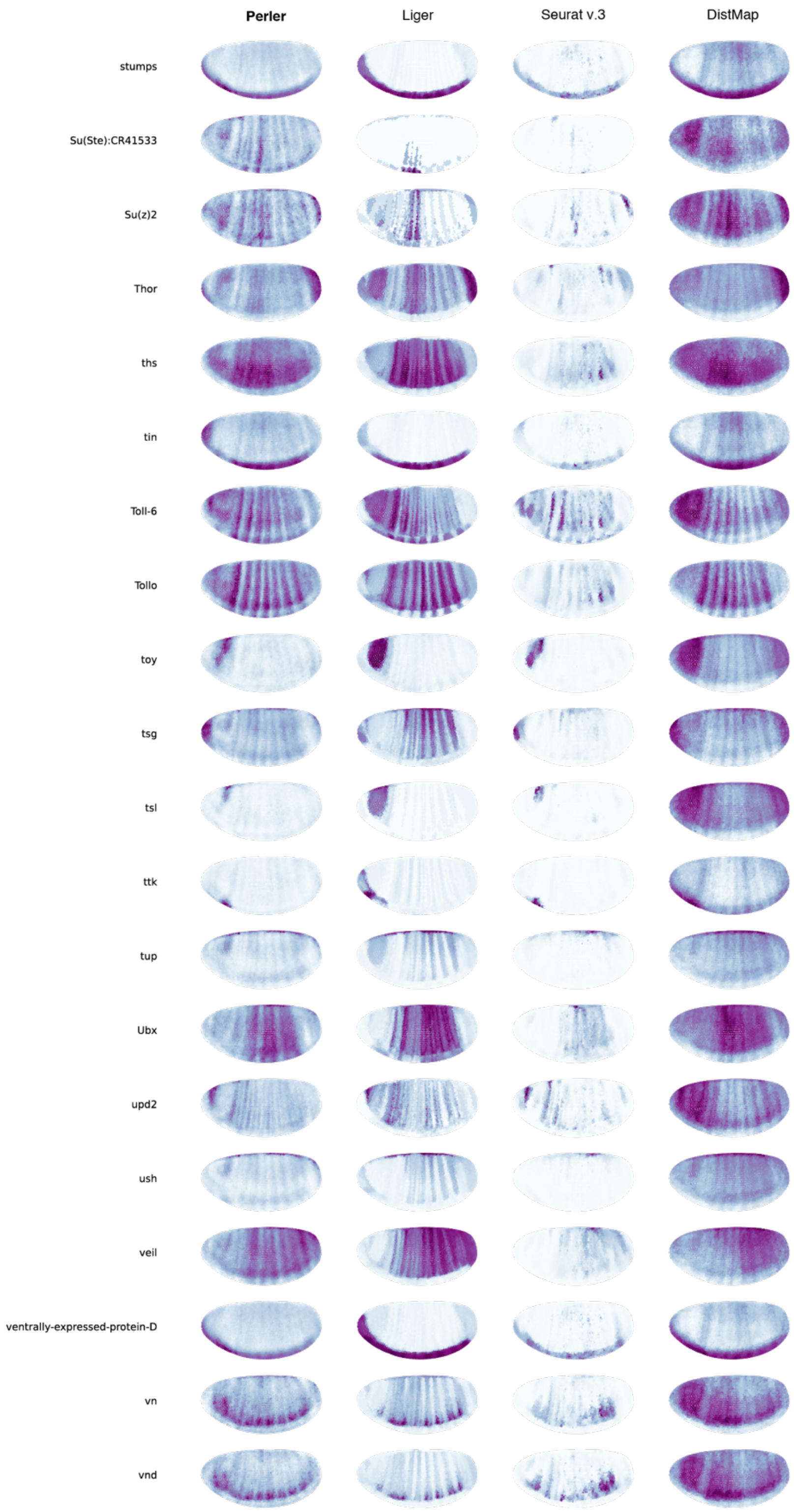

Supplementary Figure 15 (15)

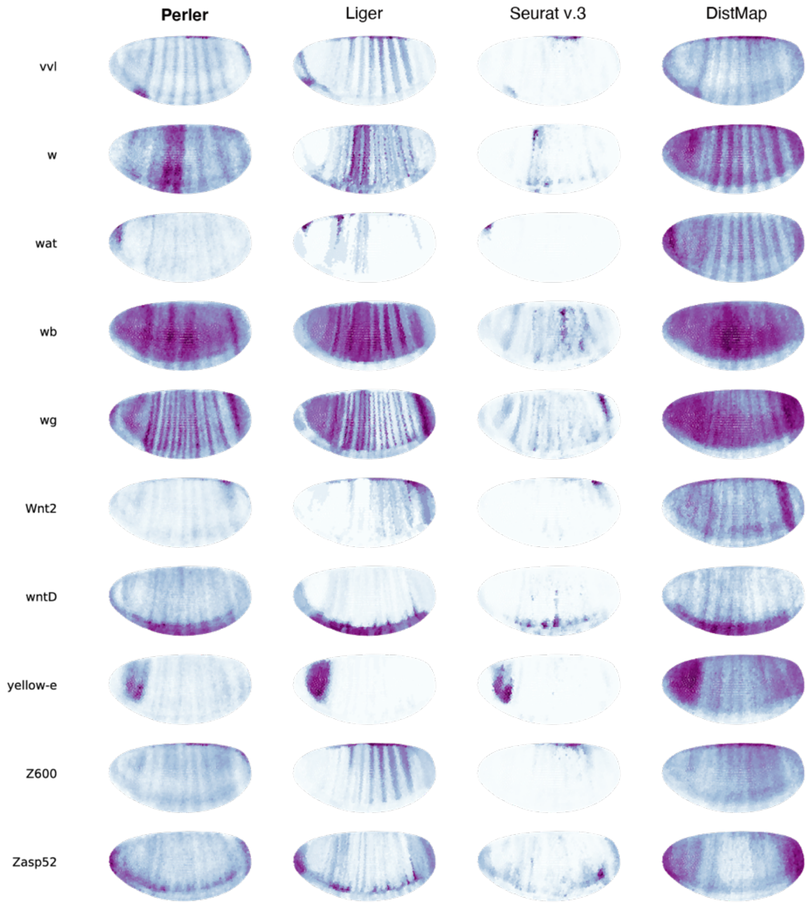

Supplementary Figure 15 (16)

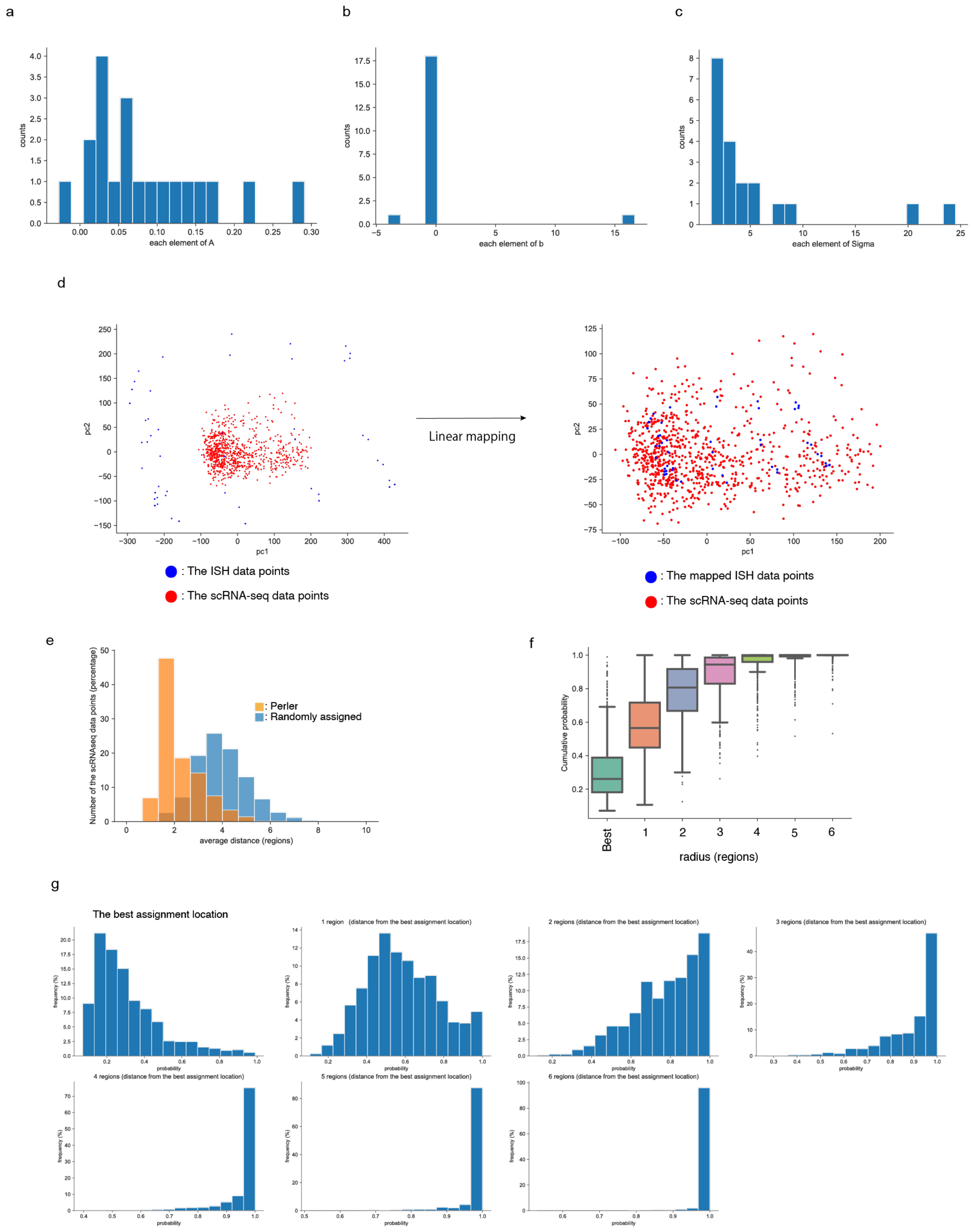
a
counts
each element of A
b
counts
each element of b
c
counts
each element of Sigma
d
pc2
pc1
Linear mapping
: The ISH data points
: The scRNA-seq data points
: The mapped ISH data points
: The scRNA-seq data points
e
Number of the scRNAseq data points (percentage)
average distance (regions)
: Perler
: Randomly assigned
f
Cumulative probability
Best
radius (regions)
g
The best assignment location
1 region (distance from the best assignment location)
2 regions (distance from the best assignment location)
3 regions (distance from the best assignment location)
4 regions (distance from the best assignment location)
5 regions (distance from the best assignment location)
6 regions (distance from the best assignment location)
frequency (%)
probability

Supplementary Figure 16: Linear mapping property of Perler in the zebrafish data

(a–c) Histograms depicting the distribution of the estimated parameters for generative linear mapping: A (left), b (middle), and Σ (right) (see Methods). Note that because A and Σ are diagonal matrices, only the diagonal elements of A and Σ are shown in the middle and right panels. (d) Scatter plot of scRNA-seq and ISH data points before (left) and after (right) mapping. Principal component analysis[14] was used to visualize high-dimensional gene-expression data into two dimensions. (e) Histogram of the assigned specificity evaluated by the distance between the optimally assigned location and the following best three locations. The distance was calculated by mean path length on the k-NN graph comprising all cells in the tissue (k = 6). (f) Boxplot of the assigned specificity (related to Figure 2d) calculated as the posterior probabilities of circular regions for each scRNA-seq data point according to radius, with the center of each region representing the optimally assigned location for each data point. For the box signifies the upper and lower quartiles, and the median is represented by a short black line within the box. The whiskers on the boxplot have a maximum 1.5 interquartile range, with black points indicating outliers. The radius was calculated by path length on the k-NN graph comprising all cells in the tissue. n=851 biologically independent cells (scRNA-seq data points). (g) Histograms of the assigned confidence corresponding to (f). Each histogram shows the detailed distributions of each boxplot in (f). Parameters of Perler are listed in Supplementary Table 7.

a

b

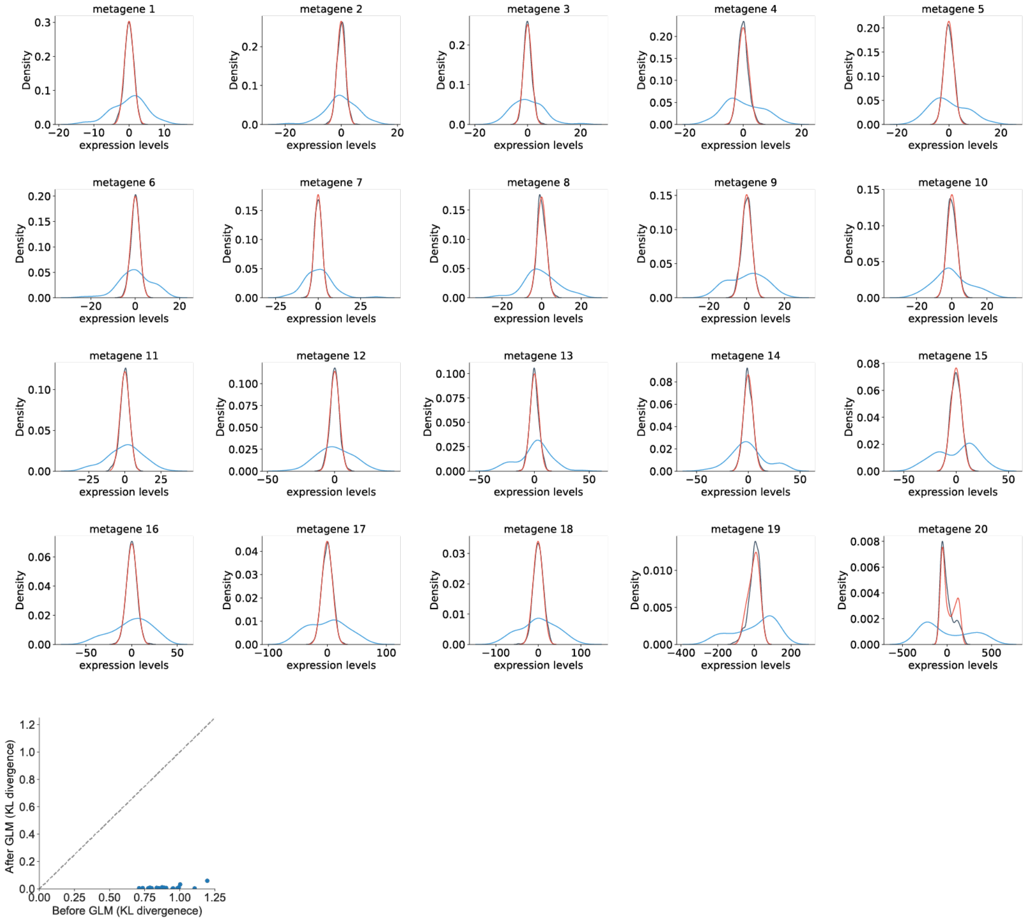

Supplementary Figure 17: Generative linear mapping on each metagene level for zebrafish data

Comparison of the distribution difference for each metagene expression level between the ISH and scRNA-seq data with those between the mapped ISH and scRNA-seq data in the zebrafish dataset. (a) Kernel density estimation of each metagene expression level in the ISH (Blue line), mapped ISH (Red line), and scRNA-seq data (Black line). For the band width parameters of the kernel density estimation in the mapped ISH data, the estimated noise parameter (c_i in equation (1)) was used. (b) Scatter plot of the distribution difference. GLM, Generative linear mapping; each dot indicates the distribution difference calculated by Kullback-Leibler divergence between the ISH or mapped ISH data and the scRNA-seq data for each metagene; grey dashed line depicts an auxiliary line showing the same Kullback-Leibler divergence before and after generative linear mapping. Parameters of Perler are listed in Supplementary Table 7.

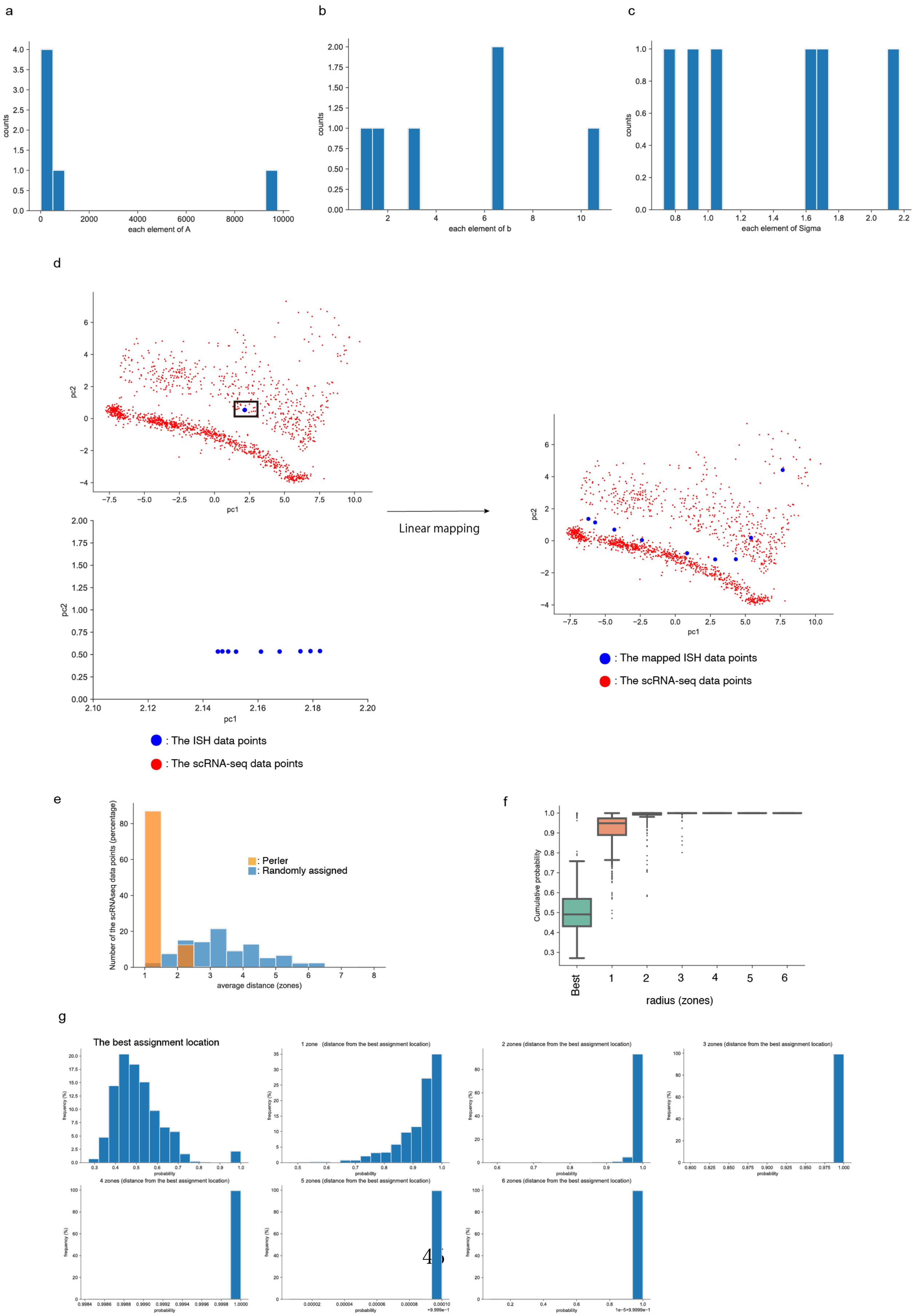
a
counts
each element of A
b
counts
each element of b
c
counts
each element of Sigma
d
pc2
pc1
Linear mapping
: The ISH data points
: The scRNA-seq data points
: The mapped ISH data points
: The scRNA-seq data points
e
Number of the scRNAseq data points (percentage)
: Perler
: Randomly assigned
average distance (zones)
f
Cumulative probability
Best
radius (zones)
g
The best assignment location
1 zone (distance from the best assignment location)
2 zones (distance from the best assignment location)
3 zones (distance from the best assignment location)
4 zones (distance from the best assignment location)
5 zones (distance from the best assignment location)
6 zones (distance from the best assignment location)
frequency (%)
probability

Supplementary Figure 18: Linear mapping property of Perler in the mammalian liver data

(a–c) Histograms depicting the distribution of the estimated parameters for generative linear mapping: A (left), b (middle), and Σ (right) (see Methods). Note that because A and Σ are diagonal matrices, only the diagonal elements of A and Σ are shown in the middle and right panels. (d) Scatter plot of scRNA-seq and ISH data points before (upper left) and after (right) mapping. Note that lower left panel depicts the enlarged panel of the upper left panel. Principal component analysis[14] was used to visualize high-dimensional gene-expression data into two dimensions. (e) Histogram of the assigned specificity evaluated by the distance between the best assigned location and the following best three locations. The distance was calculated by mean path length on the k-NN graph comprising all cells in the tissue (k = 2). (f) Boxplot of the assigned specificity (related to Figure 2d) calculated as the posterior probabilities of circular regions for each scRNA-seq data point according to radius, with the center of each region representing the best assigned location for each data point. For the box signifies the upper and lower quartiles, and the median is represented by a short black line within the box. The whiskers on the boxplot have a maximum 1.5 interquartile range, with black points indicating outliers. The radius was calculated by path length on the k-NN graph comprising all cells in the tissue. n=1415 biologically independent cells (scRNA-seq data points). (g) Histograms of the assigned confidence corresponding to (f). Each histogram shows the detailed distributions of each boxplot in (f). Parameters of Perler are listed in Supplementary Table 7.

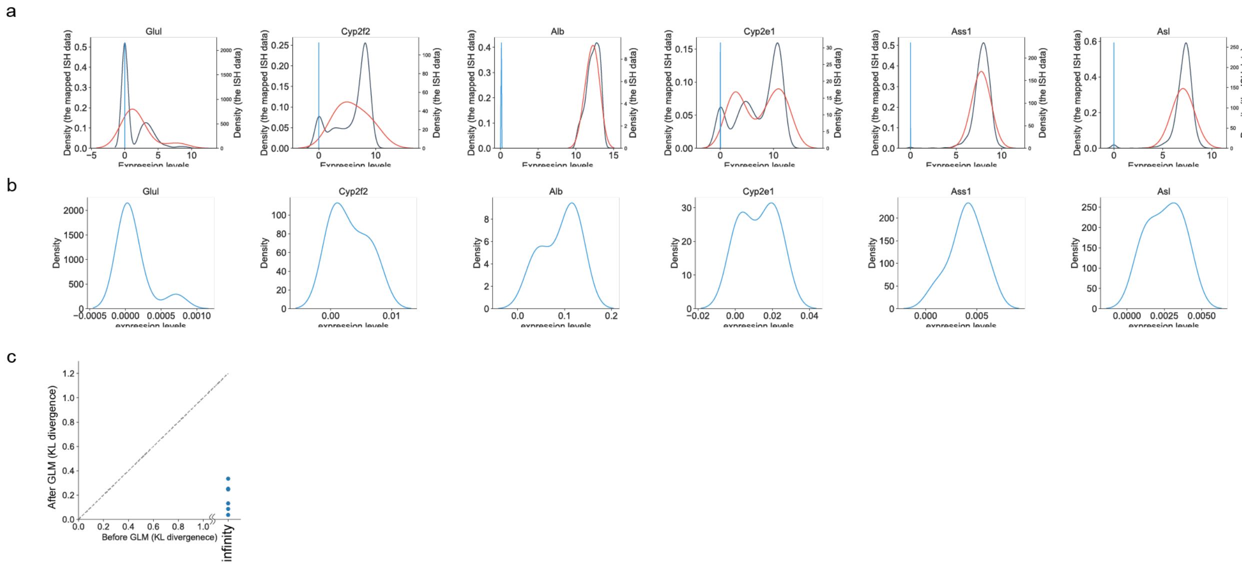

Supplementary Figure 19: Generative linear mapping on each metagene level in the mammalian liver data

Comparison of the distribution difference of each metagene expression level between the ISH and scRNA-seq data with those between the mapped ISH and scRNA-seq data in the mammalian liver dataset. (a) Kernel density estimation of each metagene expression level in the ISH (Blue line), mapped ISH (Red line), and scRNA-seq data (Black line). For the band width parameters of the kernel density estimation in the mapped ISH data, the estimated noise parameter (c_i in equation (1)) was used. (b) Enlargement of blue lines in (a). (c) Scatter plot of the distribution difference. GLM, generative linear mapping; each dot indicates the distribution difference calculated by Kullback-Leibler divergence between the ISH or mapped ISH data and the scRNA-seq data for each metagene. Note, Kullback-Leibler divergence between the ISH and scRNA-seq data before the generative linear mapping numerically diverge to infinity. Grey dashed line depicts an auxiliary line showing the same Kullback-Leibler divergence before and after generative linear mapping. Parameters of Perler are listed in Supplementary Table 7.

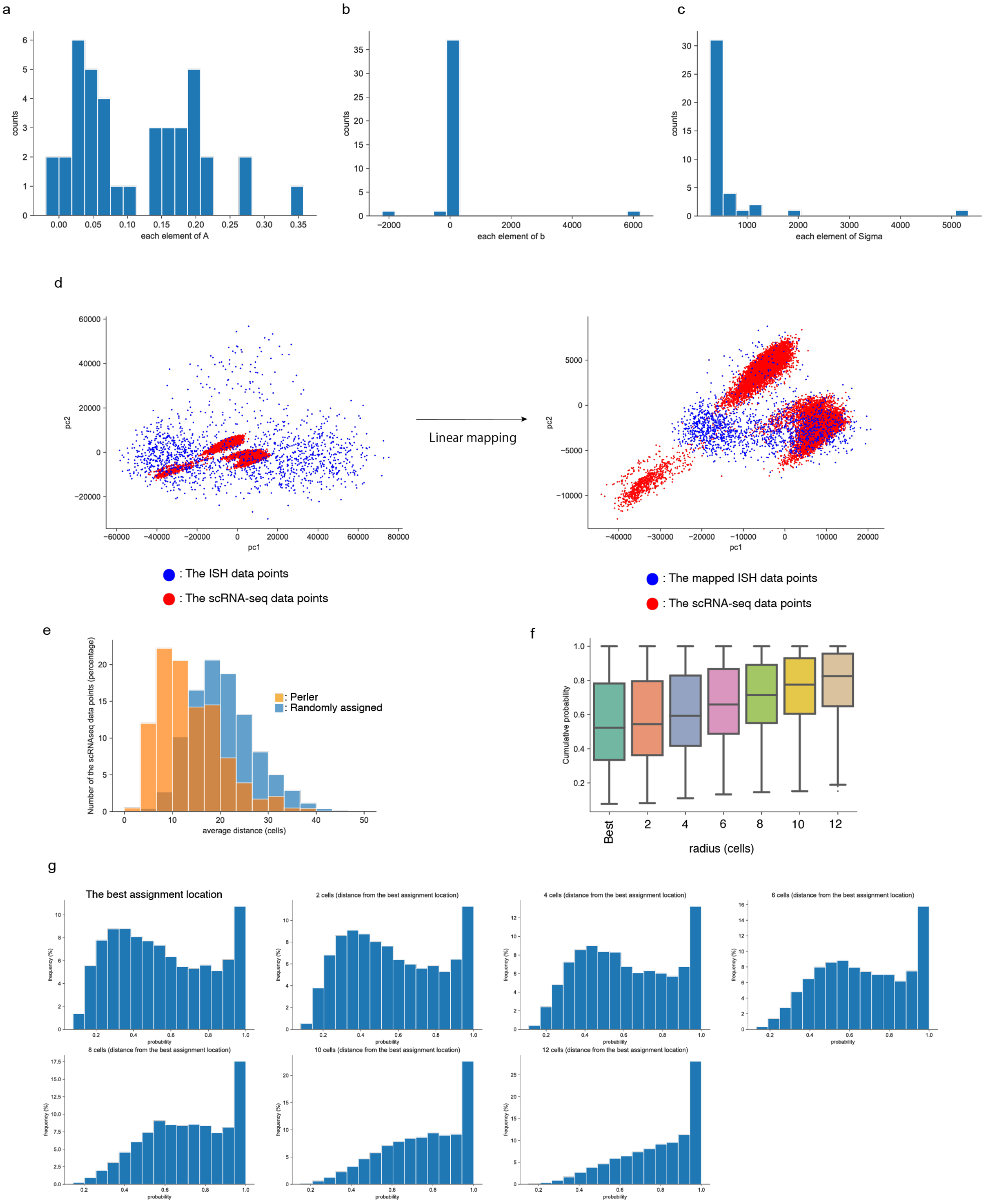
a
b
c
counts
each element of A
each element of b
each element of Sigma
d
pc1
pc2
Linear mapping
: The ISH data points
: The scRNA-seq data points
: The mapped ISH data points
: The scRNA-seq data points
e
Number of the scRNAseq data points (percentage)
average distance (cells)
: Perler
: Randomly assigned
f
Cumulative probability
radius (cells)
Best
g
The best assignment location
2 cells (distance from the best assignment location)
4 cells (distance from the best assignment location)
6 cells (distance from the best assignment location)
8 cells (distance from the best assignment location)
10 cells (distance from the best assignment location)
12 cells (distance from the best assignment location)
frequency (%)
probability

Supplementary Figure 20: Linear mapping property of Perler in the mouse cortex data

(a–c) Histograms depicting the distribution of the estimated parameters for generative linear mapping: A (left), b (middle), and Σ (right) (see Methods). Note, because A and Σ are diagonal matrices, only the diagonal elements of A and Σ are shown in the middle and right panels. (d) Scatter plot of scRNA-seq and ISH data points before (left) and after (right) mapping. Principal component analysis[14] was used to visualize high-dimensional gene-expression data in two dimensions. (e) Histogram of the assigned specificity evaluated by the distance between the optimally assigned location and the following best three locations. The distance was calculated by mean path length on the k-NN graph comprising all cells in the tissue (k = 6). (f) Boxplot of the assigned specificity (related to Figure 2d) calculated as the posterior probabilities of circular regions for each scRNA-seq data point according to radius, with the center of each region representing the optimally assigned location for each data point. For the box signifies the upper and lower quartiles, and the median is represented by a short black line within the box. The whiskers on the boxplot have a maximum 1.5 interquartile range, with black points indicating outliers. The radius was calculated by path length on the k-NN graph comprising all cells in the tissue. n=14249 biologically independent cells (scRNA-seq data points). (g) Histograms of the assigned confidence corresponding to (f). Each histogram shows the detailed distributions of each boxplot in (f). Parameters of Perler are listed in Supplementary Table 7.

a

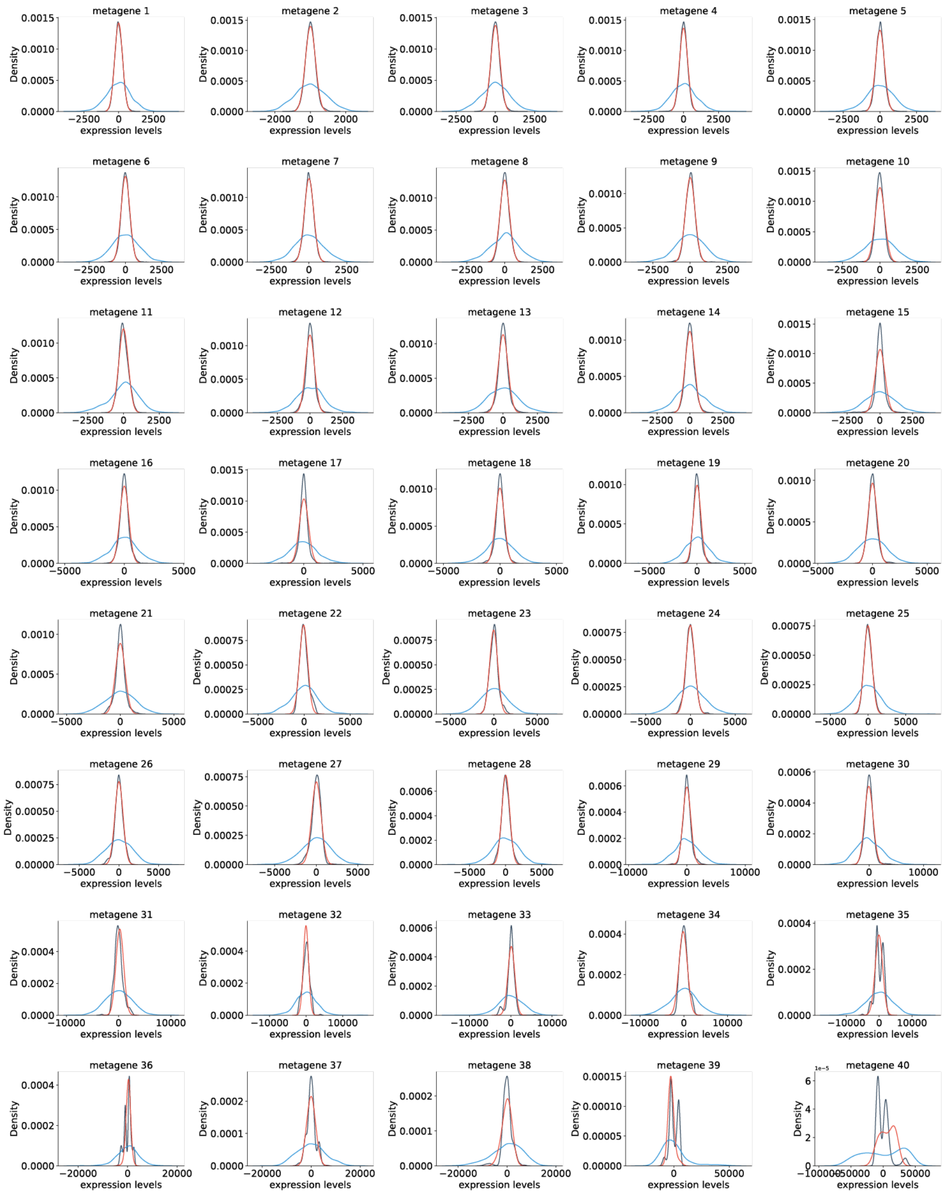
metagene 1
metagene 2
metagene 3
metagene 4
metagene 5
metagene 6
metagene 7
metagene 8
metagene 9
metagene 10
metagene 11
metagene 12
metagene 13
metagene 14
metagene 15
metagene 16
metagene 17
metagene 18
metagene 19
metagene 20
metagene 21
metagene 22
metagene 23
metagene 24
metagene 25
metagene 26
metagene 27
metagene 28
metagene 29
metagene 30
metagene 31
metagene 32
metagene 33
metagene 34
metagene 35
metagene 36
metagene 37
metagene 38
metagene 39
metagene 40
Density
expression levels

b

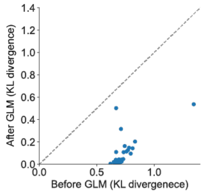
After GLM (KL divergence)
Before GLM (KL divergenece)

Supplementary Figure 21: Generative linear mapping on each metagene level for the mouse cortex data

Comparison of the distribution difference for each metagene expression level between the ISH and scRNA-seq data with those between the mapped ISH and scRNA-seq data in the mouse cortex dataset (Allen Brain Atlas data). (a) Kernel density estimation of each metagene expression level in the ISH (Blue line), mapped ISH (Red line), and scRNA-seq data (Black line). For the band width parameters of the kernel density estimation in the mapped ISH data, the estimated noise parameter (c_i in equation (1)) was used. (b) Scatter plot depicting the distribution difference. GLM, generative linear mapping; each dot indicates the distribution difference calculated by Kullback-Leibler divergence between the ISH or the mapped ISH data and the scRNA-seq data for each metagene. Grey dashed line depicts an auxiliary line showing the same Kullback-Leibler divergence before and after the generative linear mapping. Parameters of Perler are listed in Supplementary Table 7.

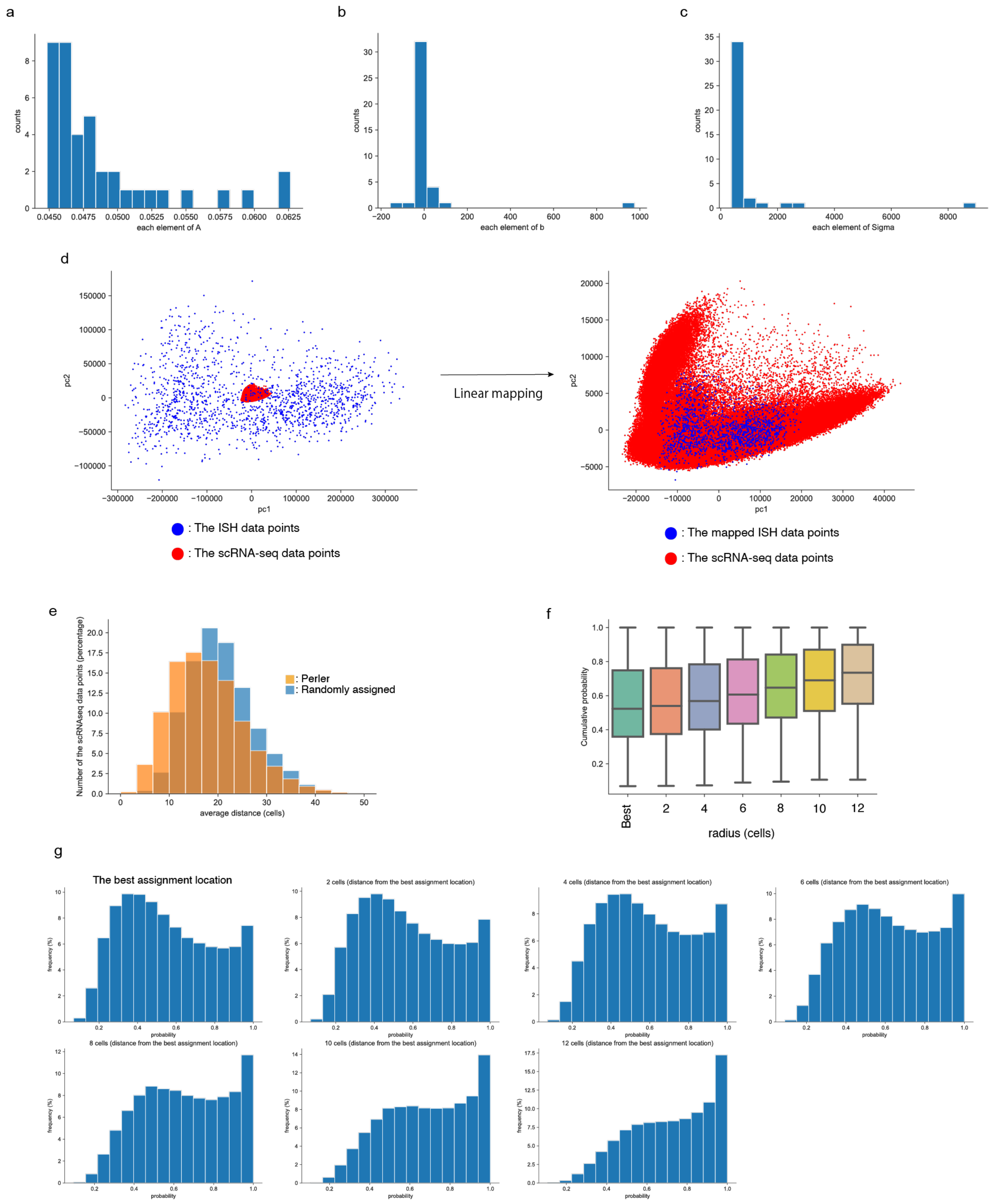
a
counts
each element of A
b
counts
each element of b
c
counts
each element of Sigma
d
pc1
pc2
Linear mapping
: The ISH data points
: The scRNA-seq data points
: The mapped ISH data points
: The scRNA-seq data points
e
Number of the scRNAseq data points (percentage)
average distance (cells)
: Perler
: Randomly assigned
f
Cumulative probability
radius (cells)
Best
g
The best assignment location
2 cells (distance from the best assignment location)
4 cells (distance from the best assignment location)
6 cells (distance from the best assignment location)
8 cells (distance from the best assignment location)
10 cells (distance from the best assignment location)
12 cells (distance from the best assignment location)
frequency (%)
probability

Supplementary Figure 22: Linear mapping property of Perler in Drop-viz data for the mouse cortex

(a–c) Histograms depicting the distribution of the estimated parameters for generative linear mapping: A (left), b (middle), and Σ (right) (see Methods). Note, because A and Σ are diagonal matrices, only the diagonal elements of A and Σ are shown in the middle and right panels. (d) Scatter plot depicting the scRNA-seq and ISH data points before (left) and after (right) mapping. Principal component analysis[14] was used to visualize high-dimensional gene-expression data in two dimensions. (e) Histogram of the assigned specificity evaluated by the distance between the optimally assigned location and the following best three locations. The distance was calculated by mean path length on the k-NN graph comprising all cells in the tissue (k = 6). (f) Boxplot of the assigned specificity (related to Figure 2d) calculated as the posterior probabilities of circular regions for each scRNA-seq data point according to radius, with the center of each region representing the optimally assigned location for each data point. For the box signifies the upper and lower quartiles, and the median is represented by a short black line within the box. The whiskers in the boxplot have a maximum 1.5 interquartile range, with black points indicating outliers. The radius was calculated by path length on the k-NN graph comprising all cells in the tissue. n=194027 biologically independent cells (scRNA-seq data points). (g) Histograms of the assigned confidence corresponding to (f). Each histogram depicts the detailed distribution of each boxplot in (f). Note, Drop-viz data is used for scRNA-seq data. Parameters of Perler are listed in Supplementary Table 7.

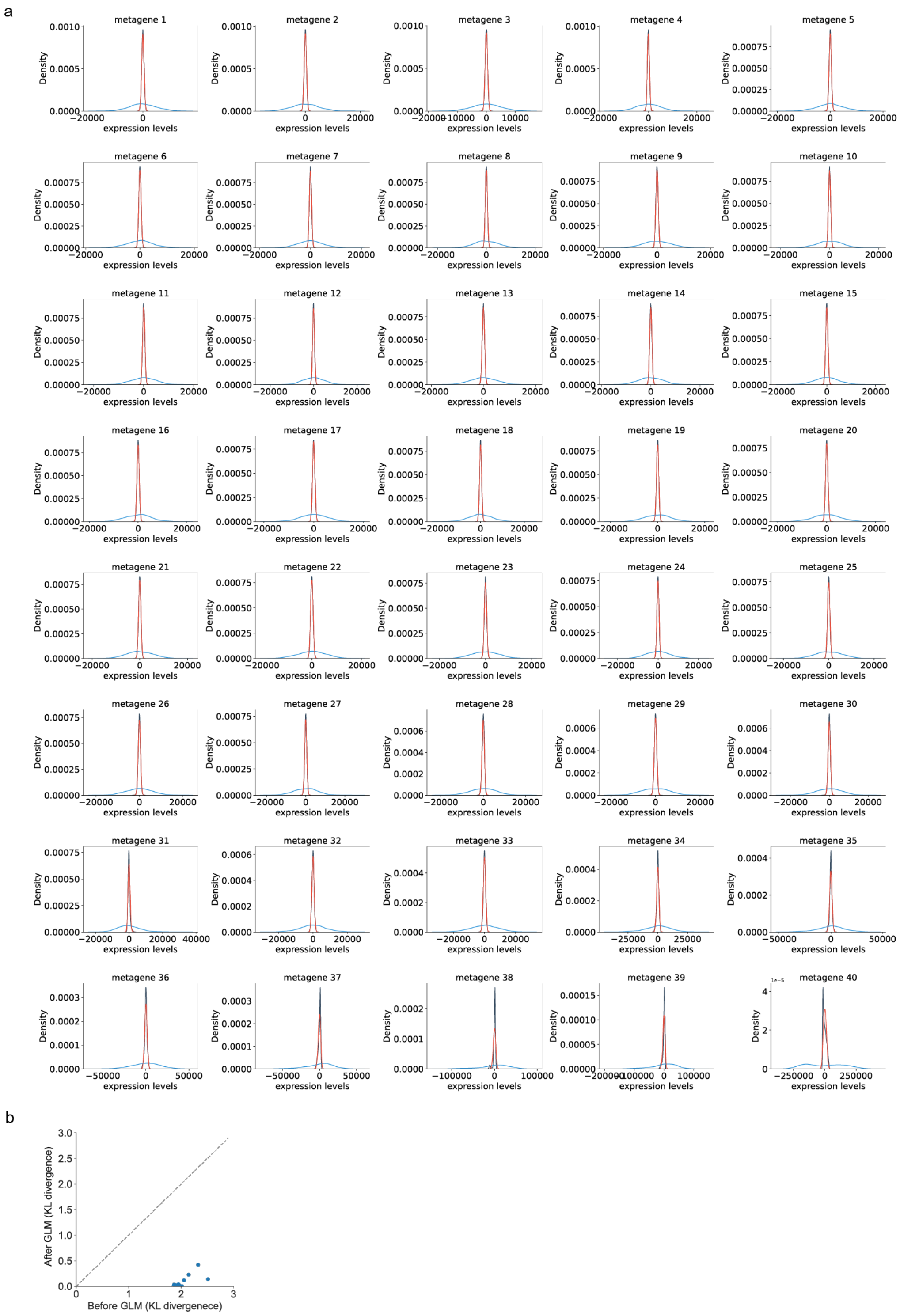
a
metagene 1
metagene 2
metagene 3
metagene 4
metagene 5
metagene 6
metagene 7
metagene 8
metagene 9
metagene 10
metagene 11
metagene 12
metagene 13
metagene 14
metagene 15
metagene 16
metagene 17
metagene 18
metagene 19
metagene 20
metagene 21
metagene 22
metagene 23
metagene 24
metagene 25
metagene 26
metagene 27
metagene 28
metagene 29
metagene 30
metagene 31
metagene 32
metagene 33
metagene 34
metagene 35
metagene 36
metagene 37
metagene 38
metagene 39
metagene 40
Density
expression levels
b
After GLM (KL divergence)
Before GLM (KL divergenece)

Supplementary Figure 23: Generative linear mapping on each metagene level in Drop-viz data for the mouse cortex

Comparison of the distribution differences for each metagene expression level between the ISH and scRNA-seq data with those between the mapped ISH and scRNA-seq data in the mouse cortex dataset (Drop-viz data). (a) Kernel density estimation of each metagene expression level in the ISH (Blue line), mapped ISH (Red line), and scRNA-seq data (Black line). For the band width parameters of the kernel density estimation in the mapped ISH data, the estimated noise parameter (c_i in equation (1)) was used. (b) Scatter plot of the distribution differences. GLM, generative linear mapping; each dot indicates the distribution difference calculated by Kullback-Leibler divergence between the ISH or mapped ISH data and the scRNA-seq data for each metagene. Grey dashed line depicts an auxiliary line showing the same Kullback-Leibler divergence before and after generative linear mapping. Parameters of Perler are listed in Supplementary Table 7.

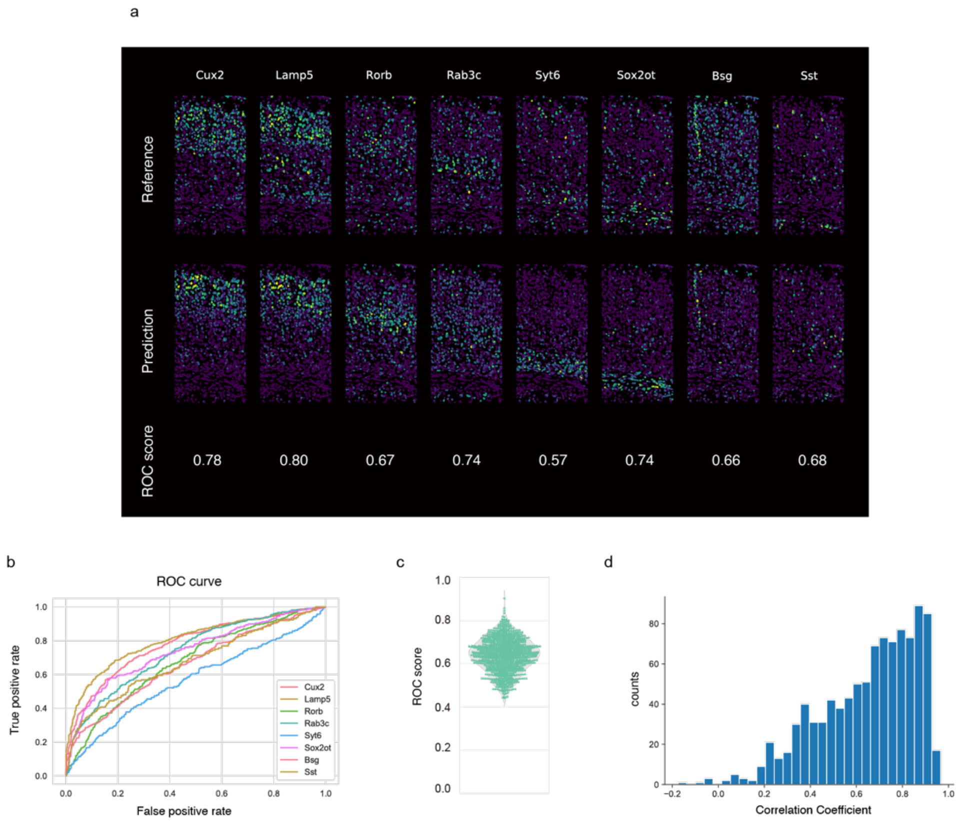

Supplementary Figure 24: Application of Perler to Drop-viz data for the mouse cortex

(a) Application of Perler to the mouse cortex (Drop-viz) data. The reference ISH data is the same as that presented in Figure 6. The upper and lower panels show the referenced ISH data and predicted gene-expression profiles, respectively. (b) ROC curve for the 10-fold CV experiments for genes shown in (a). (c) Violin plot for the predictive accuracies of Perler in the 10-fold CV experiments for all genes in the reference ISH data according to ROC score. The median ROC score is 0.64. (d) Histogram depicting correlations between gene expression predictions of Perler based on Allen Brain Atlas (Figure 6) and Drop-viz (this figure) for all landmark genes by 10-fold CV experiments. The average correlation coefficient (aCC) was 0.70. Parameters of Perler are listed in Supplementary Table 7.

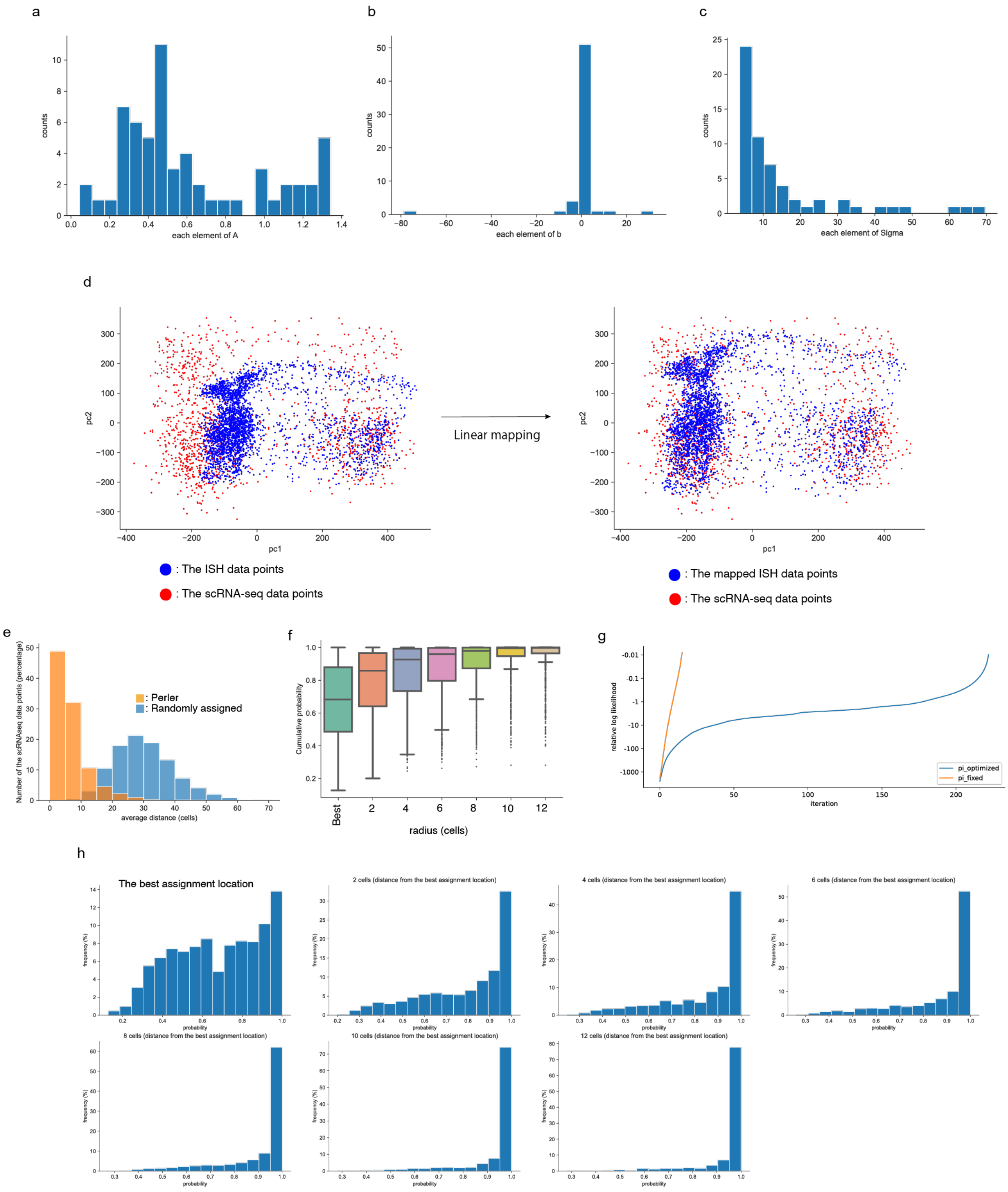
a
counts
each element of A
b
counts
each element of b
c
counts
each element of Sigma
d
pc2
pc1
Linear mapping
: The ISH data points
: The scRNA-seq data points
: The mapped ISH data points
: The scRNA-seq data points
e
Number of the scRNAseq data points (percentage)
: Perler
: Randomly assigned
average distance (cells)
f
Cumulative probability
Best
radius (cells)
g
relative log likelihood
pi_optimized
pi_fixed
iteration
h
The best assignment location
2 cells (distance from the best assignment location)
4 cells (distance from the best assignment location)
6 cells (distance from the best assignment location)
8 cells (distance from the best assignment location)
10 cells (distance from the best assignment location)
12 cells (distance from the best assignment location)
frequency (%)
probability

Supplementary Figure 25: Linear mapping property of Perler in *Drosophila* data using p_k optimization

(a–c) Histograms depicting the distributions of estimated parameters for generative linear mapping: A (left), b (middle), and Σ (right) (see Methods). Note, because A and Σ are diagonal matrices, only the diagonal elements of A and Σ are shown in the middle and right panels. (d) Scatter plot of scRNA-seq and ISH data points before (left) and after (right) mapping. Principal component analysis[14] was used to visualize high-dimensional gene-expression data in two dimensions. (e) Histogram of the assigned specificity evaluated by the distance between the optimally assigned location and the following best three locations. The distance was calculated by mean path length on the k-NN graph comprising all cells in the tissue (k = 6). (f) Boxplot of the assigned specificity (related to Figure 2d) calculated as the posterior probabilities of circular regions for each scRNA-seq data point according to the radius, with the center of each region representing the optimally assigned location for each data point. For the box signifies the upper and lower quartiles, and the median is represented by a short black line within the box. The whiskers in the boxplot have a maximum 1.5 interquartile range, with black points indicating outliers. The radius was calculated by path length on the k-NN graph comprising all cells in the tissue. n=1297 biologically independent cells (scRNA-seq data points). (g) Convergence difference of EM algorithm between analysis with and without optimization of p_k. (h) Histograms of the assigned confidence corresponding to (f). Each histogram depicts the detailed distribution of each boxplot in (f). Note that p_k was optimized in this experiment. Parameters of Perler are listed in Supplementary Table 7.

a

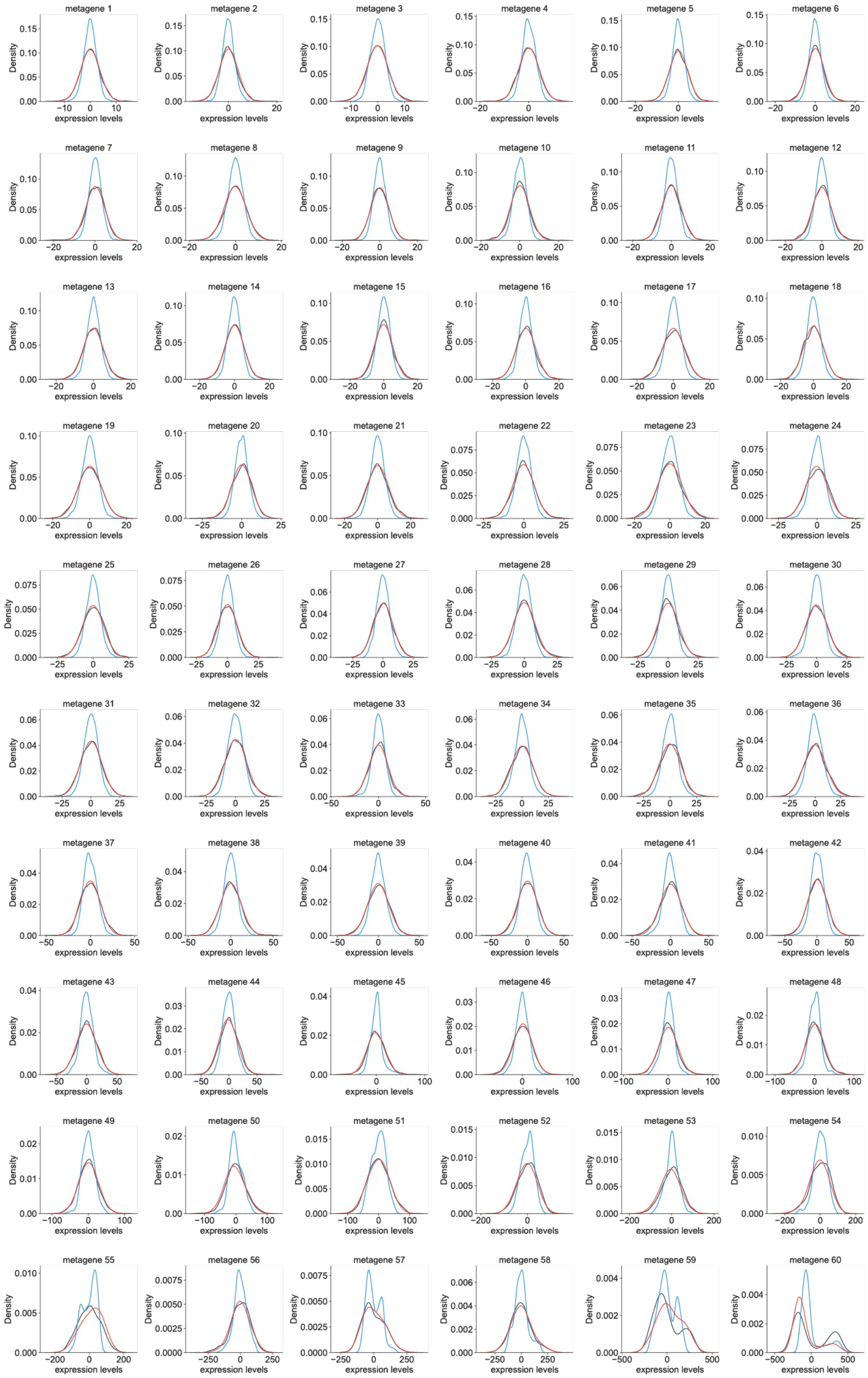
metagene 1
metagene 2
metagene 3
metagene 4
metagene 5
metagene 6
metagene 7
metagene 8
metagene 9
metagene 10
metagene 11
metagene 12
metagene 13
metagene 14
metagene 15
metagene 16
metagene 17
metagene 18
metagene 19
metagene 20
metagene 21
metagene 22
metagene 23
metagene 24
metagene 25
metagene 26
metagene 27
metagene 28
metagene 29
metagene 30
metagene 31
metagene 32
metagene 33
metagene 34
metagene 35
metagene 36
metagene 37
metagene 38
metagene 39
metagene 40
metagene 41
metagene 42
metagene 43
metagene 44
metagene 45
metagene 46
metagene 47
metagene 48
metagene 49
metagene 50
metagene 51
metagene 52
metagene 53
metagene 54
metagene 55
metagene 56
metagene 57
metagene 58
metagene 59
metagene 60
Density
expression levels

b

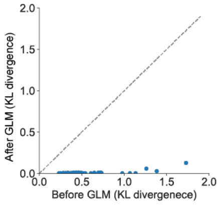
After GLM (KL divergence)
Before GLM (KL divergenece)
0.0
0.5
1.0
1.5
2.0

Supplementary Figure 26: Generative linear mapping on each metagene level for *Drosophila* data using p_k optimization

Comparison of the distribution difference of each metagene expression level between the ISH and scRNA-seq data with those between the mapped ISH and scRNA-seq data in the *Drosophila* dataset. Note that p_k was optimized in this experiment. (a) Kernel density estimation of each metagene expression level in the ISH (Blue line), mapped ISH (Red line), and scRNA-seq data (Black line). For the band width parameters of the kernel density estimation in the mapped ISH data, the estimated noise parameter (c_i in equation (1)) was used. (b) Scatter plot depicts the distribution difference. GLM, generative linear mapping; each dot indicates the distribution difference calculated by Kullback-Leibler divergence between the ISH or mapped ISH data and the scRNA-seq data for each metagene. Grey dashed line depicts an auxiliary line showing the same Kullback-Leibler divergence before and after generative linear mapping. Parameters of Perler are listed in Supplementary Table 7.

Supplementary Table 1: The well-predicted and poorly-predicted genes

Well-predicted genes	*Ama*	*Ance*	*Antp*	*apt*	*Blimp-1*	*bmm*	*bowl*	*brk*	*Btk29A*	*bun*
	cad	*CenG1A*	*CG10479*	*CG11208*	*CG17724*	*CG17786*	*CG43394*	*CG8147*	*cnc*	*croc*
	Cyp310a1	*dan*	*danr*	*Dfd*	*disco*	*Doc2*	*Doc3*	*dpn*	*ems*	*erm*
	eve	*exex*	*fkh*	*ftz*	*gk*	*gt*	*h*	*hb*	*hkb*	*htl*
	Ilp4	*ImpE2*	*ImpL2*	*ken*	*kni*	*knrl*	*Kr*	*lok*	*Mdr49*	*Mes2*
	MESR3	*mfas*	*Nek2*	*NetA*	*noc*	*nub*	*numb*	*oc*	*odd*	*prd*
	pxb	*rau*	*rho*	*run*	*sna*	*srp*	*tll*	*toc*	*Traf4*	*trn*
	tsh	*twi*	*zen*							
Poorly-predicted genes	*aay*	*CG14427*	*D*	*edl*	*Esp*	*E(spl)m5-HLH*	*fj*	*peb*	*tkv*	*zen2*
	zfh1									

Supplementary Table 2: 10-fold CV of DREAM Single-Cell Transcriptomics challenge (s1)

Comparison of Perler performance with that of Liger and Seurat v.3 using s1, one of the metrics used in DREAM Single-Cell Transcriptomics challenge[22].

	s1				
	Peler (PCA)	Perler (NA)	Seurat v.3	Liger	Christoph Hafemeister
SC1	**0.61(±0.03)**	0.59(±0.04)	0.58(±0.02)	0.52(±0.03)	0.67(±0.04)
SC2	**0.61(±0.04)**	0.59(±0.04)	0.59(±0.03)	0.51(±0.04)	0.66(±0.04)
SC3	**0.62(±0.03)**	0.61(±0.05)	**0.62(±0.04)**	0.57(±0.02)	0.61(±0.05)

SC1, 2, and 3 represent sub-challenge 1, 2, and 3 in this DREAM challenge, respectively. Each bold character indicates the optimal performance score in each sub-challenge. As a reference, the scores of Christoph Hafemeister, one of the top-ranked submissions in this DREAM challenge, are also shown. NA indicates cases without dimensionality reduction. s1 represents the correlation between the ISH expressions at the cells predicted by the proposed method and DistMap. Note, s1 was designed assuming that DistMap prediction was ground truth. For the CV scheme in Perler, we used PCA as dimensionality reduction instead of PLSC, as PLSC cannot split scRNA-seq data points into test and training data due to the singular value decomposition of the cross-covariance matrix between scRNA-seq data and ISH data (Equations (4–7)).

Supplementary Table 3: 10-fold CV of DREAM Single-Cell Transcriptomics challenge (s2)

Comparison of Perler performance with that of Liger and Seurat v.3 using s2, one of the metrics used in DREAM Single-Cell Transcriptomics challenge[22].

s2

	Peler (PCA)	Perler (NA)	Seurat v.3	Liger	Christoph Hafemeister
SC1	**1.01(±0.09)**	**1.01(±0.12)**	1.00(±0.07)	0.65(±0.06)	1.05(±0.06)
SC2	**1.00(±0.12)**	0.97(±0.14)	**1.00(±0.10)**	0.72(±0.06)	0.99(±0.08)
SC3	**0.87(±0.10)**	0.73(±0.10)	0.81(±0.11)	0.67(±0.04)	0.90(±0.07)

SC1, 2, and 3 represent sub-challenge 1, 2, and 3 in this DREAM challenge, respectively. Each bold character indicates the optimal performance score in each sub-challenge. As a reference, the scores of Christoph Hafemeister, one of the top-ranked submissions in this DREAM challenge, are also shown. NA indicates cases without dimensionality reduction. s2 represents the inverse distance of the cells predicted by the proposed method to the most probable location predicted by DistMap. Note, s2 was designed assuming that DistMap prediction was ground truth. For the CV scheme in Perler, we used PCA as dimensionality reduction instead of PLSC, as PLSC cannot split scRNA-seq data points into test and training data due to the singular value decomposition of the cross-covariance matrix between scRNA-seq data and ISH data (Equations (4–7)).

Supplementary Table 4: Ten-fold CV of DREAM Single-Cell Transcriptomics challenge (s3)

Comparison of Perler performance with that of Liger and Seurat v.3 using s3, one of the metrics used in DREAM Single-Cell Transcriptomics challenge[22].

s3

	Peler (PCA)	Perler (NA)	Seurat v.3	Liger	Christoph Hafemeister
SC1	0.55(±0.01)	**0.56(±0.01)**	0.53(±0.01)	0.37(±0.03)	0.66(±0.01)
SC2	**0.58(±0.02)**	**0.58(±0.02)**	0.55(±0.03)	0.43(±0.02)	0.70(±0.01)
SC3	**0.69(±0.02)**	0.67(±0.02)	0.68(±0.01)	0.59(±0.04)	0.64(±0.02)

SC1, 2, and 3 represent sub-challenge 1, 2, and 3 in this DREAM challenge, respectively. Each bold character indicates the optimal performance score in each sub-challenge. As a reference, the scores of Christoph Hafemeister, one of the top-ranked submissions in this DREAM challenge, are also shown. NA indicates cases without dimensionality reduction. s3 represents the gene-wise correlations between the scRNA-seq expressions of landmark genes and the ISH expressions of the most probable cell predicted by the proposed method. Note that the calculated correlations are biasedly weighted by DistMap predictability for each gene.In this metric, the genes that cannot be well predicted by DistMap are less weighted. For the CV scheme in Perler, we used PCA as dimensionality reduction instead of PLSC, as PLSC cannot split scRNA-seq data points into test and training data due to the singular value decomposition of the cross-covariance matrix between scRNA-seq data and ISH data (Equations (4–7)).

Supplementary Table 5: Comparison of Perler with existing methods

Perler characteristics relative to Liger, Seurat (v.3), DistMap, the method described by Halpern et al.[7], and Seurat (v.1).

	Perler	Liger	Seurat v.3	DistMap	Halpern et al,.	Seurat v.1
Continuous (not binary)	✓	✓	✓	×	✓	×
Applicability	✓	✓	✓	✓[b]	×	✓[b]
Dimensinality reduction	✓[a]	✓	✓	×	×	×
Generative model	✓	×	×	×	✓	✓
Linear mapping model	✓	×	×	×	×	×
Generalization	✓	×	×	×	-	-

[a]Perler used a dimensionality reduction technique (PLSC) as a preprocessing

[b]DistMap and Seurat v.1 are applicable to the datasets whose ISH data is binarized

Supplementary Table 6: Perler usage in Python code

The minimum usage of Perler. Underlined text indicates the controlled parameters that potentially affect the performance of Perler.

```
import perler
plr = perler.PERLER(data=scRNAseq, reference=ISH, n_metagenes, DR)

#Generative linear mapping (the first step of perler)
##The parameter fitting by EM algorithm
plr.em_algorithm(optimize_pi)
##Calculate the pair-wise distance between scRNAseq data and reference data
plr.calc_dist()

#Hyperparameter estimation
##conducting LOOCV experiment
##in the case that number of landmark genes are large, please use plr.k_fold_cv()
plr.loocv()
##fitting the hyperparameters by grid search
plr.grid_search()

#spatial reconstruction (the second step of perler)
plr.spatial_reconstruction(location = location)

#show results
print(plr.result_with_location.head())
```

Supplementary Table 7: Parameter used in this study

Controlled parameters are also shown in Supplementary Table 6.

Data set	n_metagenes	DR	optimize_pi	Hyperparameters
Drosophila (Fig. 2-5, Supplementary Fig. 2-9, 11, and 15)	60	PLSC	False	Optimized
Zebrafish (Fig. 6, Supplementary Fig. 10, 11, 16, and 17)	20	PLSC	False	Optimized
Mammalian liver (Fig. 6, Supplementary Fig. 11, 18, and 19)	-	-	False	Optimized
Mouse cortex (Fig. 6, Supplementary Fig. 11, 20, and 21)	40	PLSC	False	Optimized
Drosophila (Supplementary Fig. 10)	-/60	-/PLSC	False	$\alpha = 0, \beta = 1$
Zebrafish (Supplementary Fig. 10)	-/20	-/PLSC	False	Optimized
Drosophila (Supplementary Fig. 11)	60	PLSC	False	Unoptimized[a]/Optimized
Zebrafish (Supplementary Fig. 11)	60	PLSC	False	Unoptimized[a]/Optimized
Mammalian liver (Supplementary Fig. 11)	-	-	False	Unoptimized[a]/Optimized
Mouse cortex (Supplementary Fig. 11)	40	PLSC	False	Unoptimized[a]/Optimized
Drosophila (Supplementary Fig. 12 and 13)	[b]	PLSC	False	$\alpha = 0, \beta = 1$
Zebrafish (Supplementary Fig. 12 and 13)	[c]	PLSC	False	$\alpha = 0, \beta = 1$
Mouse cortex (Supplementary Fig. 12 and 13)	40	PLSC	False	$\alpha = 0, \beta = 1$
Mouse cortex (Drop-viz) (Supplementary Fig. 22-24)	40	PLSC	False	$\alpha = 0, \beta = 1$
Drosophila (Supplementary Fig. 25 and 26)	60	PLSC	True	Optimized

[a] $\alpha = \frac{1}{2}, \beta = 0$.

[b] n_metagenes are the same as the number of landmark genes.

[c] n_metagenes are the half of the number of landmark genes.

Supplementary Table 8: The computational cost of Perler

The running time and peak memory usage for the Perler procedures presented in Figure 2 and 6. Note that Perler uses multiprocessing (16 processes are used in our experiments) to accelerate the computation of the hyperparameter optimization.

Data set	Time (sec)	Memory (MiB)
Drosophila (Figure 2)	2818.53	8146.44
Zebrafish (Figure 6)	31.34	665.09
Mammalian liver (Figure 6)	397.88	1204.03
Mouse cortex (Figure 6)	3215.19	22074.47

sec, seconds; MiB, Mebibytes
